# Iron regulatory pathways differentially expressed during *Madurella mycetomatis* grain development in *Galleria mellonella*

**DOI:** 10.1038/s41467-025-60875-2

**Published:** 2025-06-25

**Authors:** Imad Abugessaisa, Mickey Konings, Ri-Ichiroh Manabe, Cathal M. Murphy, Tsugumi Kawashima, Akira Hasegawa, Chitose Takahashi, Michihira Tagami, Yasushi Okazaki, Kimberly Eadie, Wilson Lim, Sean Doyle, Annelies Verbon, Ahmed H. Fahal, Takeya Kasukawa, Wendy W. J. van de Sande

**Affiliations:** 1 Laboratory for Large-Scale Biomedical Data Technology, RIKEN Center for Integrative Medical Sciences, Yokohama City, Kanagawa Japan; 2 Premium Research Institute for Human Metaverse Medicine (WPI-PRIMe), The University of Osaka, Suita, Osaka Japan; 3 Graduate School of Medicine and Faculty of Medicine, The University of Osaka, Suita, Osaka Japan; 4 Department of Medical Microbiology and Infectious Diseases, Erasmus MC, University Medical Center Rotterdam, Rotterdam, The Netherlands; 5 Laboratory for Comprehensive Genomic Analysis, RIKEN Center for Integrative Medical Sciences, Yokohama City, Kanagawa Japan; 6 Department of Biology, Maynooth University, Maynooth, Kildare Ireland; 7 Department of Internal Medicine, University Medical Center Utrecht, Utrecht, The Netherlands; 8 Mycetoma Research Center, University of Khartoum, Khartoum, Sudan

**Keywords:** Fungal pathogenesis, Fungi, Fungal host response

## Abstract

Mycetoma is a chronic granulomatous infection of the subcutaneous tissue, most often caused by the fungal pathogen *Madurella mycetomatis*. Characteristic of the infection is the formation of grains. However, knowledge of the function and formation of the grain is limited. Here, we use a *Galleria mellonella* larvae infection model and transcriptomic profiling to identify processes associated with *M. mycetomatis* grain formation. Larvae were infected with *M. mycetomatis* and, after 4, 24, 72 and 168 h post-inoculation, RNA was extracted from larval content and sequenced. We found that 3498 *G. mellonella* and 136 *M. mycetomatis* genes were differentially expressed during infection. In particular, genes encoding proteins related to iron transport were highly expressed by both *G. mellonella* (transferrin and ferritin) and *M. mycetomatis* (SidA, SidD and SidI). LC-MS/MS analysis of *M. mycetomatis* cultured under iron-limiting conditions revealed the presence of SidA and SidD orthologs, and concurrent RP-HPLC and LC-MS identified a singly charged, putative siderophore in culture supernatant. Furthermore, we show that *M. mycetomatis* can obtain iron from holoferritin. Thus, our results highlight the importance of iron acquisition pathways during grain formation, suggesting potential avenues for development of new diagnostic and therapeutic strategies for mycetoma.

## Abstract (web summary)

Mycetoma is a chronic granulomatous infection of the subcutaneous tissue, most often caused by the fungal pathogen *Madurella mycetomatis*. Here, the authors show that iron acquisition pathways in both pathogen and host are upregulated during grain formation in an insect larva infection model.

## Introduction

Mycetoma is a neglected disease endemic in tropical and subtropical regions. It is characterized by the formation of subcutaneous tumor-like swellings and grains that spread to deep tissues and skin. The extremities are affected most, but no body part is exempted^1,2^. Grains are biofilm-like structures in which the causative agent resides^3^. The disease progression is gradual, and symptoms develop over the course of months to years. Although mycetoma can be caused by several causative agents, in more than 40% of all published cases,the fungus *Madurella mycetomatis* was the causative agent^4,5^.

Although mycetoma grain formation is one of the hallmarks of mycetoma, currently, there is little known about its function and formation. Some studies demonstrated that grain components could protect the causative organism against the host immune response and antifungal therapy^6,7^. Others have demonstrated that these grains consist of a cement-like material, mainly composed of heavy metals, melanin, lipids, and proteins^3^.

Grains can only be formed in in vivo models. The simplest model in which grain formation can be studied is the *M. mycetomatis* grain model developed in larvae of the invertebrate *Galleria mellonella*^8–10^. *G. mellonella* has been widely accepted as a model organism as the larvae are easy to use, inexpensive, have an innate immune system which is comparable to that of mammals, are free of the legal/ethical restrictions associated with the use of mammals, and most importantly the obtained results closely correlate with those obtained in mice^11–13^. In *G. mellonella, M. mycetomatis* grains can be formed, which resemble the grains in mouse models and human patients^14^. In a previous study using this model, we demonstrated that this model can be used to follow grain formation over time and that differences in protein abundance could be demonstrated by using a label-free quantitative LC-MS/MS proteomic approach^15^. However, which transcriptional responses are important in the process of grain formation are currently unknown.

In this study, we aimed to profile the transcriptomic changes of both the larval host and the *M. mycetomatis* pathogen during grain formation to enhance our understanding of grain formation.

**Fig. 1:**
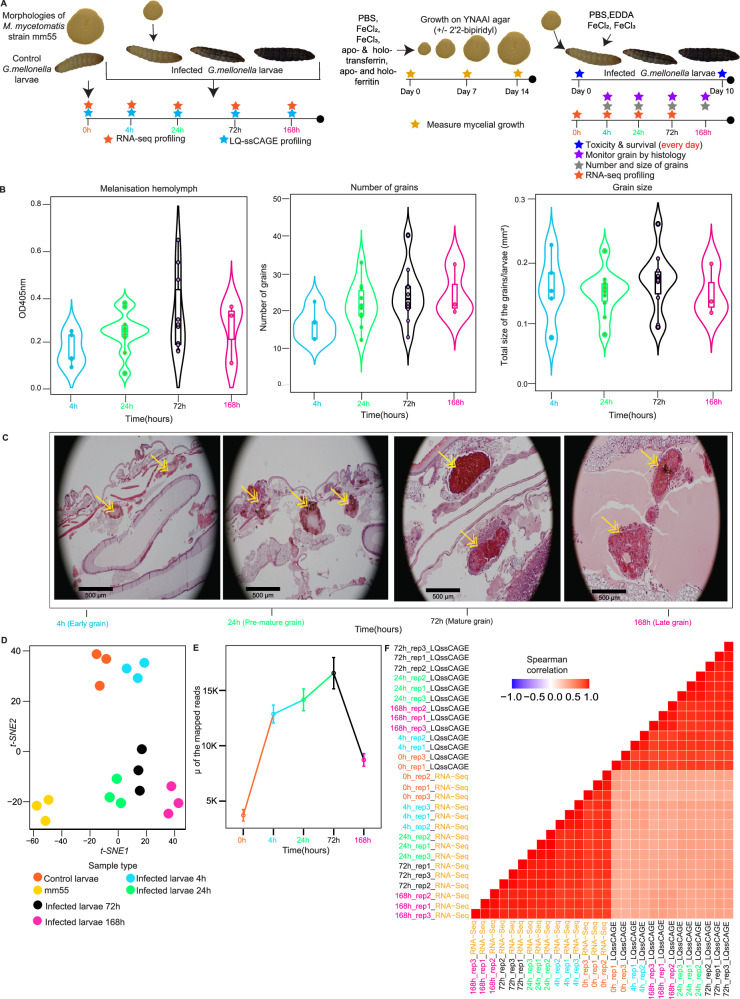
Experimental workflow, burden of infection on *G. mellonella* larvae, and quality of transcriptomics data. **A** Schematic illustration of the time course experiment design and transcriptomic assays used for high-throughput RNA profiling. Total RNA was extracted at five time points (three biological replicates each at each time point). At point 0 h, RNA was extracted from healthy *G. mellonella* larvae (host) and cultured *M. mycetomatis* strain (mm55) (pathogen) to serve as non-infected controls. After the inoculation of *G. mellonella* larvae with *M. mycetomatis*, RNA was extracted at 4, 24, 72, and 168 h post-infection (Left panel). Measure of the growth of *M. mycetomatis* cultured pathogen on YANAAI agar and YNAAI agar containing 500 M of the iron chelator 2′2-bipiridyl to study the impact of the FeCl_2_, FeCl_3_, apo- and holo-transferrin, and apo- and holoferritin on the growth (Middle panel). Impact of iron on the infection in the model. Grain development was monitored for 10 days, and different measures were performed. Total RNA was extracted at time 0, 4, 24, and 72 h, and RNA-Seq performed (Right panel). **B** To evaluate the burden of infection in the host, the melanisation of the hemolymph, the number of grains, and the grains size were measured and plotted in violin plots for each time point. In each of the violins, the box indicates the interquartile range (first quartile(Q1), third quartile(Q3), and the median value). The highest values are noted at *t* = 72 h (the maximum burden of infection). Non-infected larvae were included as controls. The data shown is an example based on observations from three evaluated biological replicates. The difference in the total number of grains or the total grain size observed on the respective time points was determined using the Mann–Whitney *U* test in GraphPad Prism 8. A *p* value > 0.05 was deemed significant (Methods). **C** Grain formation in the host was visualized 40 times magnified using H&E staining and light microscopy. Yellow arrows point towards the grain inside the capsule. Based on the characteristics of the grain development observed at each time point, the grains were defined as early, pre-mature, mature, and late grains. In the early grains, the cement material is not formed, and hemocytes are present between hyphae. In the pre-mature grains, hemolymph is forming, but still, individual hemocytes can be noted within the forming cement material. In the mature grain, the cement material is completely formed, and a capsule surrounds the grain. In the late grain, you see the capsule disappearing and an influx of hemocytes towards the grain. **D** t-*SNE* clustering: Host and pathogen RNA-Seq libraries mapped to *G. mellonella* genome assembly ASM364042v2. The replicates at each time point are shown as colored dots. The t-*SNE* plot shows the clustering of the RNA-Seq libraries mapped to the host genome. **E** Pathogen transcripts detected in host RNA-Seq after infection (three biological replicates were used at each time point, total *n* = 15). The line plot shows the number of pathogen reads detected in the host RNA-Seq data after infection, the largest number of the pathogen transcripts detected at time 72 h (mature grain) (lines are colored by time point). Data in the line plot are represented as mapped sequenced reads ± SEM. **F** RNA-Seq vs. LQ-ssCAGE mapped sequence reads correlation. The correlation matrix shows the Spearman correlation of the RNA-Seq and LQ-ssCAGE reads between replicates. Source data are provided as a Source Data file (**B**–**E**). Source data are available in the Gene Expression Omnibus (GEO) under accession numbers GSE213329 and GSE213332(**F**).

We infected *G. mellonella* larvae with *M. mycetomatis*, extracted total RNA at 0 h (healthy larvae), 4, 24, 72, and 168 h post-infection (Fig. 1A, left), and prepared RNA-Seq and Low Quantity Single Strand CAGE (LQ-ssCAGE) libraries^16^ and profiled their transcriptomic responses at each time point. We found differentially expressed pathways in both the larval host and *M. mycetomatis*, among which we identified the interplay in iron regulation between host and pathogen. To mechanistically confirm the findings from the transcriptomic profiling about iron regulation, we first established that *M. mycetomatis* could obtain iron from holoferritin in the presence of iron chelator 2′2-bipiridyl (Fig. 1A, middle). To validate siderophore biosynthesis by *M. mycetomatis* under iron-limiting conditions, we performed LC-MS/MS analysis, which reveals the presence of large number of mycelial proteins responsible for ornithine hydroxylation and fusarinine C biosynthesis. Furthermore, we performed RP-HPLC analysis of pathogen culture supernatants under iron-limiting conditions and with the addition of Fe^3+^ to the culture. Finally, we analyzed the influence of changing the iron conditions in vivo using our experimental model and analyzed the survival of *G. mellonella* exposed to different iron-influencing conditions (Fig. 1A, right).

The findings about siderophore biosynthesis pathways in *M. mycetomatis* and the influence of iron concentrations on mycetoma grain formation will impact future research for developing novel diagnostic and therapeutic strategies for eumycetoma.

The high-quality multi-Omics datasets generated from our in vivo and in vitro experiments are rich resources for future investigations and can be used to interrogate various processes important in grain formation.

## Results

### Maximum burden of infection observed 3 days after inoculation

Based on the grain size, the total number of grains, and the melanisation of the hemolymph, the maximum burden of the infection was observed 72 h after infection (Fig. 1B). All larvae died within 192 h after infection (Supplementary Fig. 1). During infection, mycetoma grains developed over time. Early grains, which were small in size and loosely structured, were observed at 4 h post-inoculation. After 24 h, more mature grains were observed. The hyphae formed within the grain, and the immune cells (hemocytes) were present in the cement material. At the maximum burden of infection, mature grains were present and surrounded by a capsule and immune cells (Fig. 1C). In the late grain, present at 168 h, disintegration of both the capsule and grain was observed, and immune cells were entering the grain.

### Pathogen transcripts detected in the grain

We noted that 88.8% of the total RNA-Seq reads in the grains originate from the *G. mellonella* larvae (Host), while only 0.01% of the RNA-Seq reads originate from *M. mycetomatis* (Pathogen) (Supplementary Data 1). The rest of the RNA-Seq reads (11.19%) are unmapped reads. Likewise, for LQ-ssCAGE, we found that 31.6% of the reads are mapped to the *G. mellonella* larvae, while only 0.66% reads mapped to the *M. mycetomatis* (Supplementary Data 1). This was not unexpected. It has already been demonstrated that in a typical mycetoma grain, only roughly 10% of *M. mycetomatis* DNA was found, the remaining DNA originated from the host^17^.

The t-*SNE* clustering of the host and pathogen transcripts mapped to the *G. mellonella* genome shows strong clustering of the samples per each time point and indicates the quality of the transcriptomics profiles (Fig. 1D and Supplementary Fig. 2). The three samples of the cultured *M. mycetomatis* pathogen (yellow dots) cluster separately from the rest of the samples (host). The *G. mellonella* reads mapped to the pathogen genome indicate the presence of the conserved reads between the two species (host and pathogen) and the presence of pathogen reads after infection (Fig. 1E). RNA-Seq and LQ-ssCAGE expression levels of *G. mellonella* larvae genes positively correlated across all samples, with a median Spearman’s correlation (0.51) (Fig. 1F).

### *G. mellonella* differentially expressed genes (DEGs) exhibited grain development stage-specific expression

**Fig. 2:**
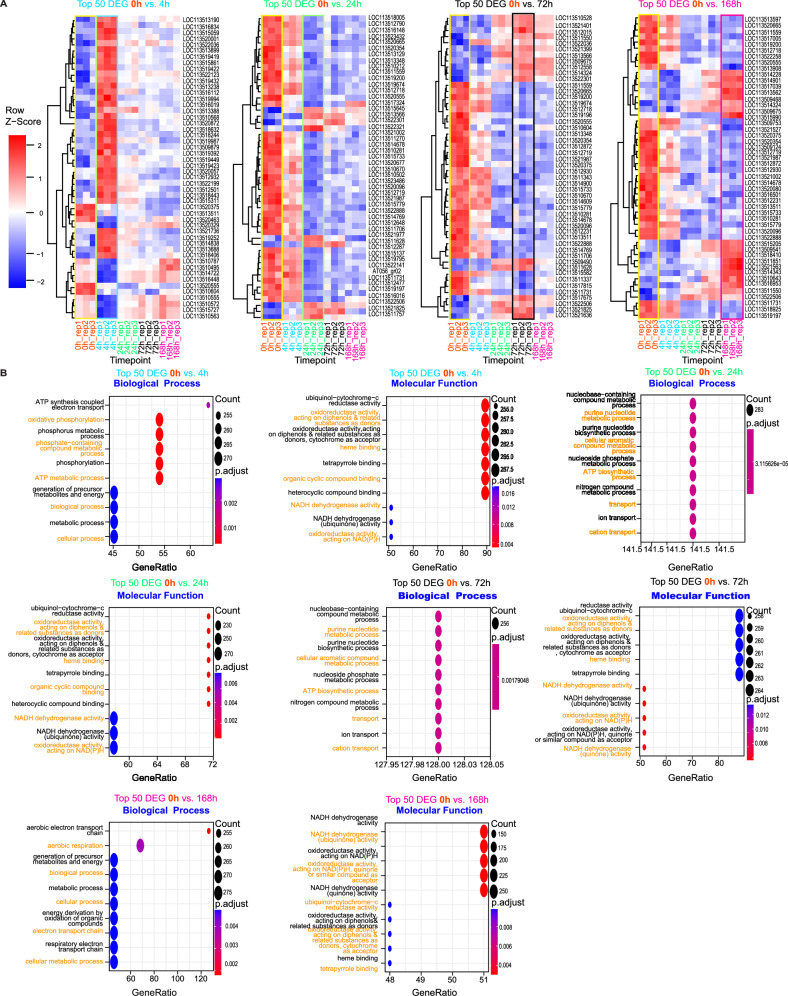
Hierarchical clustering of the host top DEGs and gene set enrichment analysis. **A** Heatmaps showing the hierarchical clustering of the top 50 DEGs of *G. mellonella* for four consecutive changes (Methods). Columns represent the biological replicates (3 replicates per time point), and rows represent the host gene symbols. Gene names are provided (Supplementary Data 5). Replicates are colored and grouped per time point. 0 h replicates are highlighted with yellow rectangles. Heatmap cells are colored by *Z*-score scale (−2 to 2). **B** Dotplots showing enriched pathways in biological processes and molecular functions in the host during infection. Each pair of plots shows enriched pathways of the top DEGs at each consecutive point. Each pathway is represented as a dot. The dots are colored by the *p-adjusted* value as computed by the gseGO function of the clusterProfiler R package (pvalueCutoff parameter of gseGO was set to <0.05). For testing differentially expressed genes, *F*-statistic, the associated *P* value, *adj* *P* value were corrected using Benjamini–Hochberg multiple testing correction (Statistics and reproducibility). The count represents the number of genes that belong to a given gene set, and the GeneRatio represents the count/setSize. setSize is the total number of genes in the gene set. Each pathway corresponds to a Gene Ontology (GO) term. Source data are available in the Gene Expression Omnibus (GEO) under accession number GSE213329.

From the time course transcriptomic profile and analysis using the RNA-Seq data (Methods), we observed that DEGs manifested in eumycetoma grain development stage-specific expression. We investigated two types of DEGs. First, the sharp changes, which detect the changes between pre-infection and post-infection. Second, the consecutive changes, which detect the changes between consecutive time points, and between every time point and 0 h (Methods). To understand the patterns of the expression of the DEGs per each time point (grain development stage), we performed hierarchical clustering of the top 50 DEGs. The clustering shows changes of expression of the top 50 DEGs (Fig. 2A), at 0 vs. 4 h, most of the genes become upregulated at 4 h. In 0 vs. 24 h, most of the genes become downregulated after 24 h. Similar patterns were observed for 0 vs. 72 h and 0 vs. 168 h. Likewise, we observed the changes in DEGs in the rest of the time points 4 h vs. all, 24 h vs. all, and 72 vs. 168 h (Supplementary Fig. 3). Validation by RT-qPCR of selected differentially expressed genes with known functions, in the infected *G. Mellonella* libraries, confirmed their expression in these samples (Supplementary Data 2 and Supplementary Fig. 4).

The UpSet diagram in Supplementary Fig. 5A summarizes the consecutive changes in DEG. For the sharp change category, 4338 genes are differentially expressed between the healthy and infected larvae (Supplementary Fig. 5B). We summarized the DEGs in all consecutive changes and the sharp changes. We found that 3575 genes are differentially expressed (Supplementary Fig. 5C). The Volcano plot in Supplementary Fig. 6 summarizes the *p* values and fold change of the DEG per time point.

### Gene set enrichment analysis of the top *G. mellonella* DEGs

To understand the biological meaning of the DEGs, we performed a gene set enrichment analysis of the top DEG (Methods). The enrichment analysis shows that several DEGs are significantly enriched in biological processes and molecular functions (Fig. 2B). Several GO terms are enriched at each time point. At 0 vs. 4 h, most of the enriched GO terms are linked to oxidative phosphorylation (GO:0006119), ATP metabolic process (GO:0046034), energy derivation by oxidation of organic compounds (GO:0015980), generation of precursor metabolites and energy (GO:0006091). All these terms are linked to energy pathways. The 0 h vs. 24 and 0 h vs. 72 were mostly enriched in the nucleobase metabolic process, purine nucleotide biosynthetic process, and ion and cation transport. Among the latter, genes linked to iron homeostasis, such as melanotransferrin, cytochrome b561-like isoforms, aconitate hydratase, and proton-coupled folate transporters, were differentially expressed. At the late-stage grain development (0 vs. 168 h), mostly enriched GO terms are related to aerobic electron transport chain and energy pathways. The full list of the top 50 DEGs and the associated annotation are in Supplementary Data 3.

### *M. mycetomatis* genes are differentially expressed in infected *G. mellonella* larvae

**Fig. 3:**
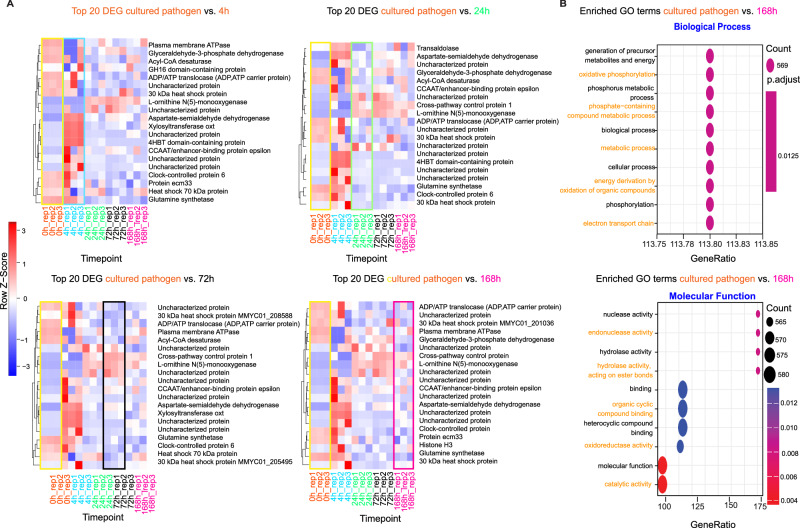
Pathogen reads in infected host and gene set enrichment analysis. **A** Heatmaps of the top 20 DEG cultured pathogen vs. pathogen reads in infected host 4 h. Full annotation of the genes in each of the heatmaps in (Supplementary Data3). **B** GSEA of the top 20 DEG (For testing differentially expressed genes, *F*-statistic, the associated *P* value, and the *adj.* *P* value were corrected using Benjamini–Hochberg multiple testing correction.) pathogen genes and enriched GO terms (gseGEO parameter pvalueCutoff = 0.05). For testing differentially expressed genes, *F*-statistic, the associated *P* value, *adj.* *P* value were corrected using Benjamini–Hochberg multiple testing correction (Statistics and reproducibility). Source data are available in the Gene Expression Omnibus (GEO) under accession number GSE213329.

To study the presence and expression of the pathogen genes in infected *G. mellonella* larvae, we mapped all samples to the *M. mycetomatis* genome assembly ASM127576v2 (Methods). We observed the same time-patterns of expression as in the expression of the host DEGs, when we clustered the top 20 pathogen DEGs (Fig. 3A). Genes which appeared to be differentially expressed at all time points include Glyceraldehyde-3-phosphate dehydrogenase, 30 kDa heat shock protein and ECM33, for which the corresponding proteins were previously demonstrated to be differentially abundant by LC-MS/MS^15^. Complete annotation of the pathogen top 20 DEGs is provided in Supplementary Data 4. The GSEA of the top DEG of the pathogen is shown in Fig. 3B, with several GO terms enriched.

### Host and pathogen transcript dynamics and response patterns

**Fig. 4:**
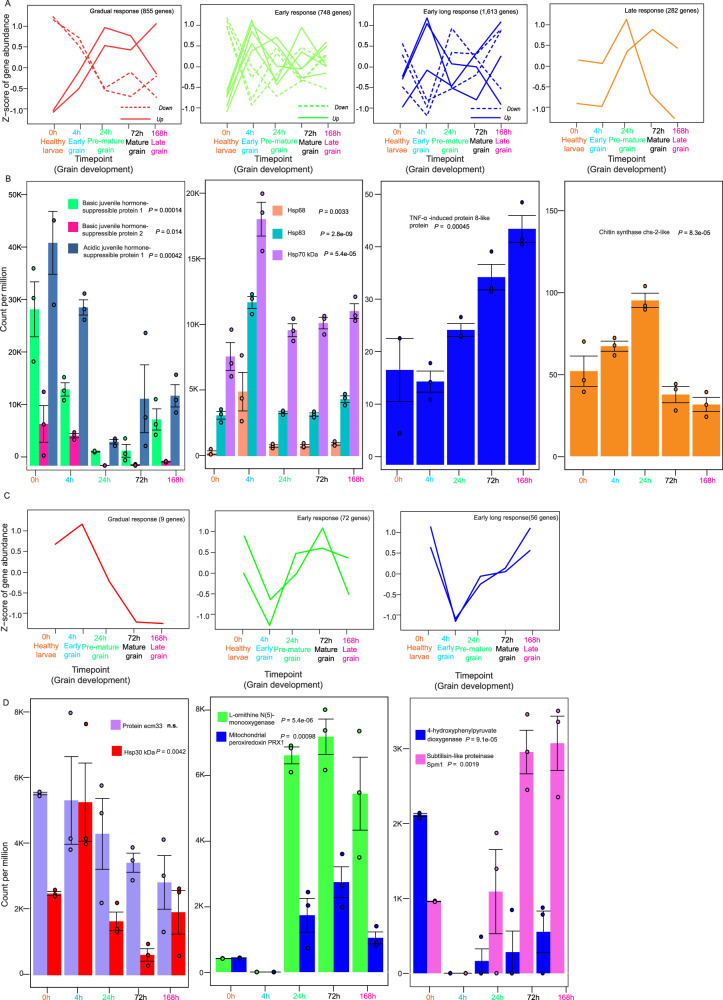
Host and pathogen transcriptomic changes before and after the development of Eumycetoma grain. **A** The responses of host DEGs are shown in four patterns (Gradual, early, early long, and late response). *Y*-axis represents the *Z*-score of gene abundance, and the *X*-axis shows the time point and grain development stage. The gradual, early response and early long response show sub-patterns (down and up) shown as dashed or continuous lines, respectively. Full annotation of the genes in each response group in (Supplementary Data 6). **B** Expression of some of the host DEGs corresponding to the patterns in (**A**). *Y*-axis shows the count per million (CPM), and the *X*-axis shows the time point (*n* = 15, 3 biological replicates per time point). Data are presented as mean values ± SEM. **C** Transcriptomic response patterns of the pathogen DEGs. Only three patterns are detected. As in (**A**), *Y* and X axes represent the *Z*-score gene abundance and grain development stage, respectively. Full annotation of the genes in each response group is in Supplementary Data 8. **D** Expression of some of the pathogen DEGs corresponding to the patterns in (**C**) (*n* = 15, 3 biological replicates per time point). Data are presented as mean values ± SEM. We computed a two-way ANOVA test. Source data for (**A**, **C**) are available in the Gene Expression Omnibus (GEO) under accession number GSE213329. Source data for (**B**, **D**) are provided as a Source Data file.

Since we ended up with high number of DEGs from the RNA-Seq data, we further investigated the dynamics of the expression and response pattern of the host and pathogen transcriptome to the infection separately (Fig. 4A). Of the 3498 host DEGs, 3252 genes have shown very distinct temporal dynamics. We observed a set of (1006:3252) host genes that start to gradually respond to the infection by either upregulation or downregulation after 0 h. We classified those genes as gradual response genes. The second set of (697:3252) DEGs were classified as early response genes, which immediately change the expression pattern upon infection. Most of the genes in this category change their expression either up or down at 24 h. The third set of (1488:3252) DEGs were the early-long response patterns in which the immediate change in transcription at 4–24 h remains longer during infection. The last pattern of the response was the late response genes, these (61:3252) genes start to change their expression at 24 h. Figure 4B shows an example of a gene for each expression pattern category. Detailed annotation of genes in each response pattern was obtained from the UniProt database (Supplementary Data 5)^18^. For the pathogen genes, we observed all the above patterns as in the host, except the late response pattern genes. Of the total 136 pathogen DEGs, only nine genes gradually change expression after the infection (Fig. 4C). Interestingly, among these genes, Ecm33 and the stress-related hsp30 were noted^19^. Proteomic data demonstrated that protein Ecm33 was present in the eumycetoma grain at all time points analyzed^15^. Bar plots in Fig. 4D demonstrate the expression of example genes. Detailed annotation of the 136 pathogen genes with corresponding GO term annotation is listed in (Supplementary Data 6).

### Genes involved in iron sequestering are differentially expressed and contribute to eumycetoma grain development

To investigate the biological relevance of the differentially expressed genes, we performed Protein-Protein Interaction (PPI) network enrichment analysis of relevant genes. We first decided to focus on biological processes known to be expressed by *M. mycetomatis*. We demonstrated that melanisation of the grains occurs rapidly during the course of infection, and some of the genes involved in the melanin biosynthesis pathway were differentially expressed (up or down regulated) (e.g., 4-hydroxyphenylpyruvate, Fig. 4D). Based on this information, we expected the complete biosynthesis pathway for DHN-melanin and pyomelanin to be expressed during infection. For DHN-melanin and pyomelanin, NCBI BLAST^20^ found significant similar homologous in *M. mycetomatis* for all genes. We identified all homologs of *A. fumigatus* DHN-melanin and pyomelanin biosynthesis pathways in *M*. *mycetomatis* using the NCBI BLAST^20^ web service (Supplementary Data 7). Supplementary Fig. 7 shows the DHN-melanin biosynthesis pathway and pyomelanin biosynthesis pathway in *A. fumigatus* and the PPI networks of homologous genes in *M. mycetomatis*. These PPIs indicate the functional interaction link between genes involved in both the DHN-melanin and pyomelanin biosynthesis pathway and other genes in *M. mycetomatis* with a PPI network enrichment *p* value of 3.08E-13 and 0.00388, respectively. The list of homologs of *A. fumigatus* DHN-melanin and pyomelanin in *M. mycetomatis* with their statistical report from NCBI BLAST^20^ is listed in Supplementary Data 7.

One of the main findings of the DEG analysis was significant expression changes in genes related to heme-binding and iron regulation. Therefore, we decided to further analyze these regulatory pathways for both *G. mellonella* and *M. mycetomatis*.

**Fig. 5:**
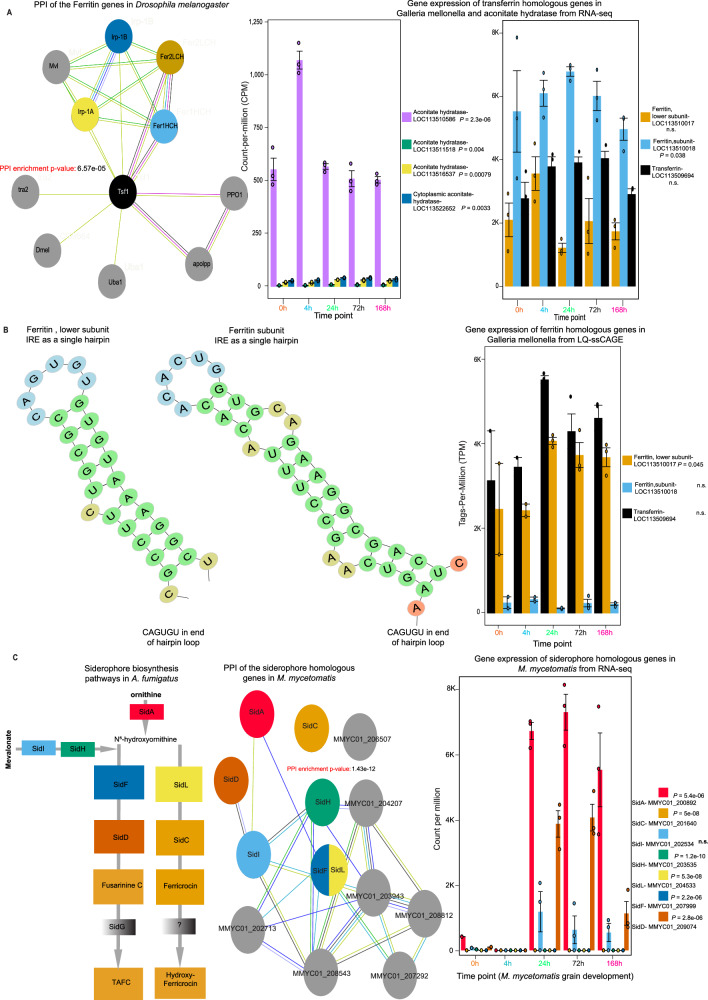
Iron regulation in *G. mellonella* and siderophore biosynthesis pathways in *M. mycetomatis.* **A** PPI of Ferritin genes in *Drosophila melanogaster* adapted from G. Xiao et al., with a focus on transferrin (Tsf1), Ferritin 1 Heavy Chain Homologue (Fer1HCH), Ferritin 2 Light Chain Homologue (Fer2LCH), Iron-regulatory protein 1A and 1B (Irp-1A and Irp-1B)^62^. Expression levels of respective *G. mellonella* homologs during infection are shown in the bar plot. Aconitate hydratase encoding genes LOC113511518, LOC113516537, LOC113522652, and LOC113510586 are differentially expressed during infection (three biological replicates were used at each time point, total *n* = 15). Data are presented as mean values ± SEM. **B** Generated hairpin loops in LOC113510017 and LOC113510018 transcripts, containing the conserved CAGUGU sequence characteristic for Iron-Responsive Elements (IREs). Expression of ferritin and transferrin homologous genes in *G. mellonella* based on LQ-ssCAGE (*n* = 15, 3 biological replicates per time point). In the barplot, the error bar represents the standard error of the mean (SEM). **C** Siderophore biosynthesis pathway adapted from Gründlinger et al.^28^. The pathway starts with mevalonate converted to anhydromevalonyl-CoA by SidI and SidH^29^. *A. fumigatus* uses three hydroxamate-type siderophores for iron uptake. The extracellular triacetylfusarinine C (TAFC), hyphal ferricrocin (FC), and conidial hydroxyferricrocin (HFC)^29^. N⁵-hydroxyornithine is generated from ornithine by SidA. The SidG gene has no homologs gene in *M. mycetomatis*, and the gene marked as (?) is not characterized yet in *A. fumigatus*. In the PPI, SidF and SidL are annotated to the same gene in *M. Mycetomatis* (N(6)-hydroxylysine O-acetyltransferase). No significant similarity was found for SidG in *M. mycetomatis*. All siderophore genes except SidG are differentially expressed in RNA-Seq data, as shown in the bar plot (*n* = 15, 3 biological replicates per time point). Data are presented as mean values ± SEM. We computed a two-way ANOVA test; the data in the bar chart are represented as expression ± SEM. Source data for the bar plots are provided as a Source Data file.

To investigate the iron regulation response of *G. mellonella* during infection, we focused on genes encoding for expression of ferritin and transferrin, proteins responsible for the transport and storage of iron^21^. The list of homologs involved in iron regulation in *D. melanogaster* and *G. mellonella* with their statistical report from NCBI BLAST^20^ is listed in Supplementary Data 8. We constructed a PPI network, indicating the functional interaction of iron-regulatory genes in *D. melanogaster* (Fig. 5A). With a PPI network enrichment *p* value of (0.00312), strong functional interaction is indicated between the following genes: Ferritin 1 Heavy Chain Homologue (Fer1HCH), Ferritin 2 Light Chain Homologue (Fer2LCH), Transferrin (Tsf1), Iron-regulatory protein 1A (Irp-1A), Iron-regulatory protein 1B (Irp-1B), and Malvolio (Mvl). Except for Mvl, homologs of the respective genes were identified in *G. mellonella*, and expression levels are depicted in the bar chart (Fig. 5A). The genes ferritin subunit (LOC113510018), ferritin lower subunit (LOC113510017), transferrin (LOC113509694), aconitate hydratase (LOC113516537/ LOC113511518) and cytoplasmic aconitate hydratase (LOC113522652), are all consistently expressed in both healthy and infected *G. mellonella* larvae over time. This data is in line with previous literature in which mRNA expression of ferritin in healthy *G. mellonella* larvae is detected, and mRNA expression of transferrin is detected in both healthy larvae and infected larvae of *Mandunca sexta*^22,23^. In contrast with a proteomic analysis of healthy *G. mellonella* larvae and larvae infected with *M. mycetomatis*, we did not observe differential expression of both ferritin and transferrin coding genes^15^.

**Fig. 6:**
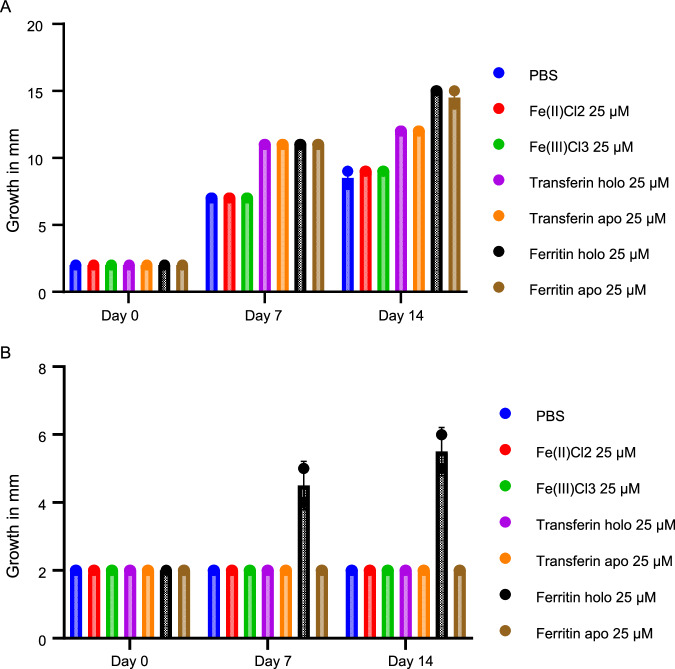
Growth of *M. mycetomatis* measured by the diameter of the mycelium. **A** Growth of *M. mycetomatis* on YNAAI agar supplemented with PBS, FeCl_2_, FeCl_3_, apo- and holo-transferrin, and apo- and holoferritin. Two biological replicates were performed. Data presented as mean value, showing both individual data points. **B** Growth of *M. mycetomatis* on YNAAI agar containing 500 M of the iron chelator 2′2-bipiridyl, and supplemented with PBS, FeCl_2_, FeCl_3_, apo- and holo-transferrin, and apo- and holoferritin. Two biological replicates were performed. Data presented as mean value, showing both individual data points. Source data are provided as a Source Data file.

Gene expression does not always directly correlate with protein expression. Therefore, we postulated that the presence of one or multiple translational regulatory mechanisms might explain the difference between the proteomic and transcriptomic findings. This is strengthened by our PPI analysis and gene expression data (Fig. 5A), showing the expression of multiple aconitate hydratases (Irp-1A and Irp-1B), genes encoding for iron-regulatory proteins which regulate translation by binding to iron-responsive elements (IRE) in the 5′ UTR region^24^. Of the multiple aconitate hydratases, homologs expressed, LOC113511518, LOC113516537, LOC113522652, and LOC113510586 were differentially expressed (Fig. 5A). To further validate the functional similarity with known iron-regulatory processes, we identified the IRE regions present in mRNA transcripts. These IRE regions manifest in the form of a hairpin loop, containing a conserved CAGUGU at the end of the hairpin loop. Figure 5B shows the identified IRE hairpin structures of the ferritin subunit and ferritin lower subunit in the 5′ UTR region. The IRE of the lower ferritin subunit was identical to the one earlier described for the 32 kDa *G. mellonella* subunit, while the IREs for the upper ferritin subunit differed in a few amino acids from the one described for the 26 kDa ferritin subunit^25,26^. LQ-ssCAGE data reveals expression activity of the promoter regions for the ferritin subunit (LOC113510018), ferritin lower subunit (LOC113510017), and transferrin (LOC113509694) (Fig. 5B), indicating that these regions were actively expressed. Altogether, this data supplements the proteomic findings, suggesting the presence of an iron-regulatory mechanism in *G. mellonella* for the overexpression of ferritin to store intracellular iron molecules during *M. mycetomatis* infection. In response to the withdrawal of iron from haemolymph by *G. mellonella, M. mycetomatis* needs to alter its iron regulation. We next looked through the top 20 DEG for *M. mycetomatis*, L-ornithine N(5)-monooxygenase (homologs to SidA) and nonribosomal peptide synthetase SidD were identified (Fig. 3A), which are key in the siderophore biosynthesis pathway found in *A. fumigatus* and *A. nidulans* (Fig. 5C)^27,28^. NCBI BLAST^20^ found significantly similar homologs in *M. mycetomatis* for all genes except SidG (Supplementary Data 7). PPI analysis of relevant genes for the *M. mycetomatis* homologs was performed. The PPI network analysis (Fig. 5C) revealed a strong interaction between the expressed *M. mycetomatis* homologs for the entire siderophore biosynthesis as described for *A. fumigatus*^28^. The PPI network obtained with enrichment *p* value (1.83E-14) showed no interaction between SidC and the rest of the network. In the PPI network (Fig. 5C), the top enriched biological processes (Gene Ontology) are GO:0006635 (Fatty acid beta-oxidation), GO:0006631 (Fatty acid metabolic process), GO:0003995 (acyl-CoA dehydrogenase activity), and GO:0003857 (3-hydroxyacyl-CoA dehydrogenase activity). The top KEGG pathways enriched in the PPI network are map01100 (Metabolic pathways) FDR (1.11E-05) and map00071 (Fatty acid degradation) FDR (0.0024). Both Acyl-CoA ligase sidI and Mevalonyl-coenzyme A hydratase sidH are the third-degree hub nodes in the PPI network (Fig. 5C). In *A. fumigatus*, SidI and SidH link biosynthesis of mevalonate and triacetylfusarinine C (TAFC)^29^. Seven genes found to have functional link in the PPI network in (Fig. 5C) (gray nodes), these genes are MMYC01_206507 (5′-hydroxyaverantin dehydrogenase), MMYC01_204207 (3-hydroxyisobutyryl-CoA hydrolase), MMYC01_203943 (Short/branched chain specific acyl-CoA dehydrogenase), MMYC01_208812 (Glutaryl-CoA dehydrogenase), MMYC01_202713 (3-hydroxybutyryl-CoA dehydrogenase), MMYC01_207292 (Acetylglutamate kinase), and MMYC01_208543 (3-hydroxybutyryl-CoA dehydrogenase). All siderophore homologous genes in *M. mycetomatis* are differentially expressed. We observed that L-ornithine N(5)-monooxygenase (SidA), Acyl-CoA ligase (SidI), and Nonribosomal peptide synthase (SidD) are highly expressed in all time points with significant upregulation 1 day after the infection compared to the rest of the genes involved in the siderophore pathway (Fig. 5C). In response to the withdrawal of iron from hemolymph by *G. mellonella*, *M. mycetomatis* needs to alter its iron regulation. We therefore first established that *M. mycetomatis* could obtain iron from holoferritin in the presence of iron chelator 2′2-bipiridyl (Fig. 6).

### Validation of siderophore production by *M. mycetomatis*

**Fig. 7:**
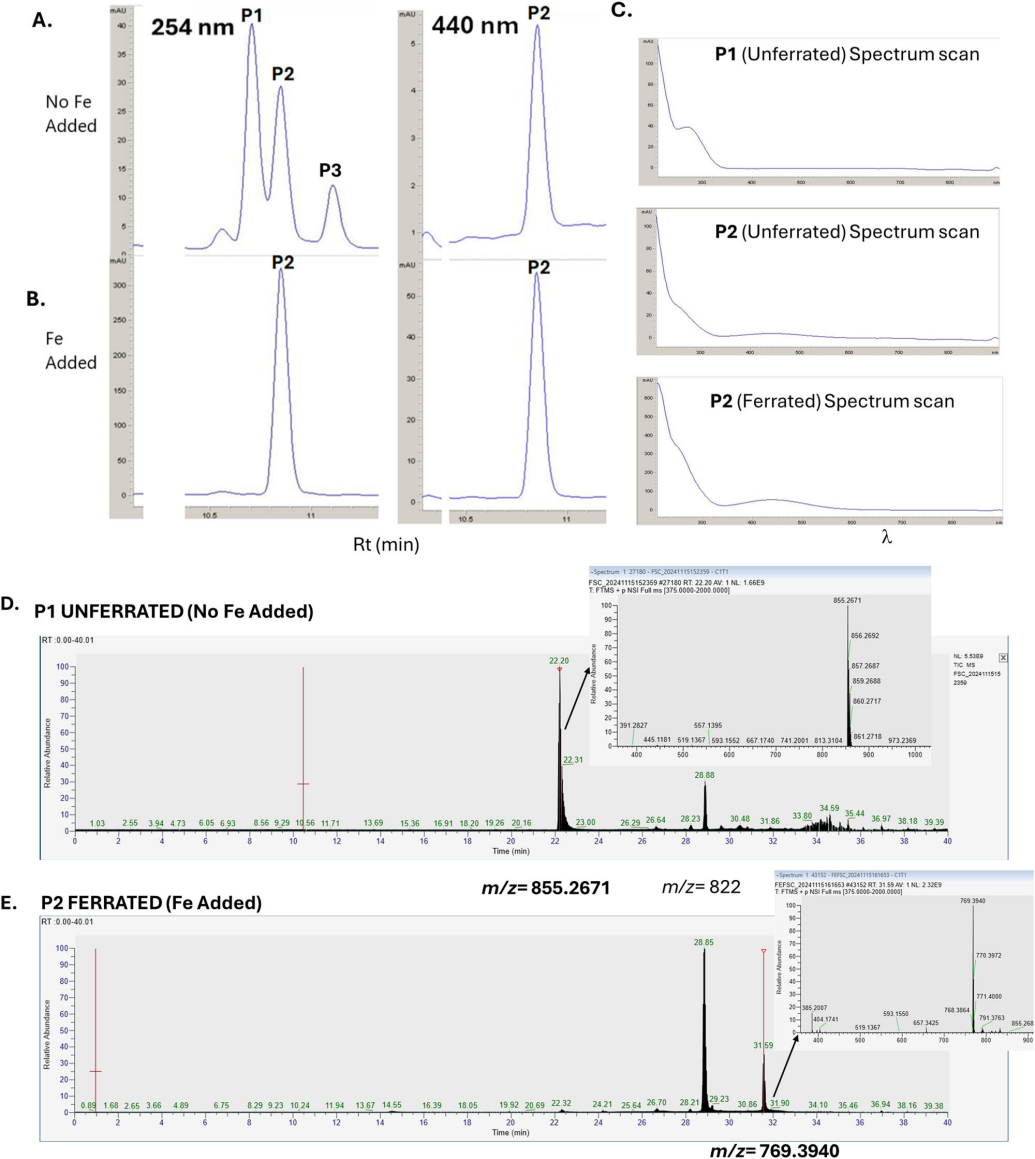
Validation of *M. mycetomatis* siderophore biosynthesis. **A** RP-HPLC analysis identified the time-dependent presence of three secreted metabolites (P1–P3) in untreated culture supernatants at A254 nm. P2 also showed weak A440 nm. **B** Post-ferration RP-HPLC revealed that P1 shifted to co-elute with P2 (increased A 254 nm and a 10-fold increase in 440 nm). **C** Spectrum scanning confirmed increased A440 + 20 nm, specifically following ferration for P2. **D** High-resolution LC-MS analysis of unferrated P1 (Rt 22.2 min) revealed a singly charged, putative siderophore with *m*/*z* = 855.2671 (inset) and a compound with *m*/*z* 822. **E** Since the species with *m*/*z* = 855.2671 disappeared upon Fe^3+^ addition, it represents a hitherto unidentified *M. mycetomatis* siderophore. Compound with *m*/*z* 822 is also evident in P2 (ferrated), and the nature of the species with *m*/*z* 769.3940 (inset) remains to be revealed. Source data are available via the PRIDE partner repository with the dataset identifier PXD063449.

LC-MS/MS analysis of *M. mycetomatis* cultured under iron-limiting conditions revealed the presence of 1,099 mycelial proteins, including SidA (57.4 kDa) (23% sequence coverage) and SidD (439.7 kDa) (2.5% sequence coverage) orthologs, which are responsible for ornithine hydroxylation and fusarinine C biosynthesis, respectively, in *A. fumigatus* (Supplementary Fig. 8A, B). Concurrent RP-HPLC analysis of culture supernatants identified the time-dependent secretion of 3 metabolites (P1–P3) with A254 nm, one of which (P2) showed weak A440 nm (Fig. 7A). This culture supernatant was also reactive in the Siderotec^TM^ siderophore detection assay (data not shown). Following Fe^3+^ addition to culture supernatant, repeat RP-HPLC revealed that P1 shifted to co-elute with P2 as indicated by increased A254 nm and a 10-fold increase in 440 nm (Fig. 7B). This is highly diagnostic of Fe^3+^ binding and siderophore activity. Associated spectral analysis revealed low specimen Abs (400–450 nm) without ferration but increased Abs 400–450 nm following ferration (Fig. 7C). In addition, high-resolution LC-MS analysis of unferrated P1 revealed a singly charged, putative siderophore (Rt: 22.2 min) *m*/*z* = 855.2671 (Fig. 7D). Since this species disappeared upon Fe^3+^ addition (Fig. 7E), it likely represents a key *M. mycetomatis* siderophore.

### The influence of changing the iron conditions

**Fig. 8:**
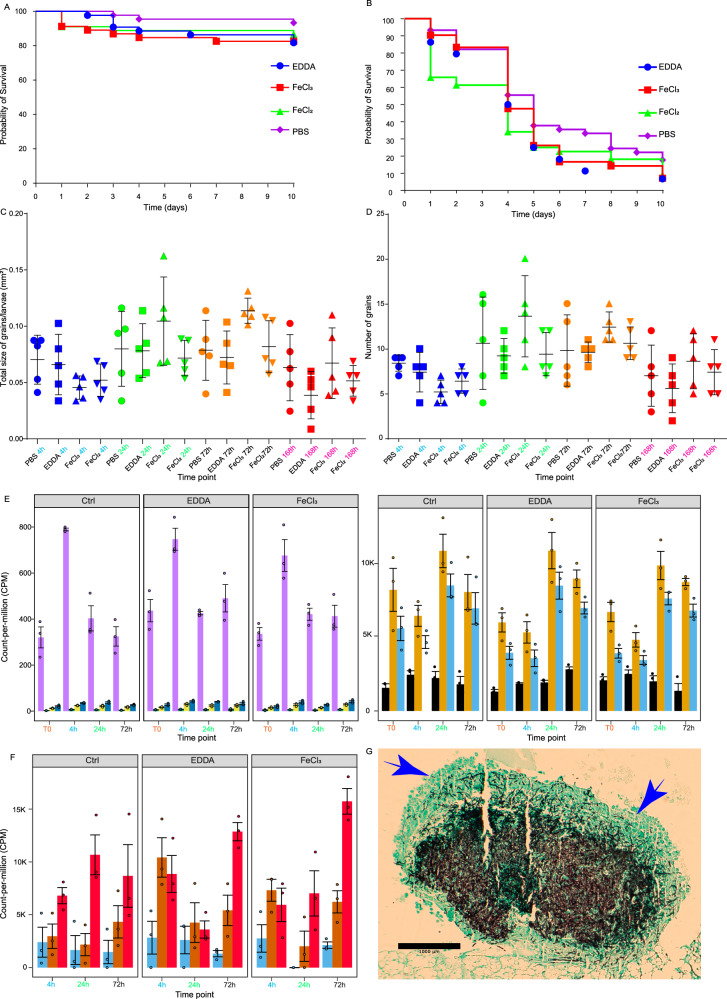
Effect of iron on the infection in *G. mellonella* larvae. **A** Toxicity of 332 µM EDDA, and 25 µM FeCl_2_ and FeCl_3_ in *G. mellonella*. **B** Survival of *M. mycetomatis*-infected larvae upon exposure to 332 µM EDDA, 25 µM FeCl_2_, and 25 µM FeCl_3_. **C** *M. mycetomatis* grain count in *G. mellonella* larvae upon exposure to 332 µM EDDA, 25 µM FeCl_2_, and 25 µM FeCl_3_. Data are presented as mean values ± SD, determined based on five biological replicates. **D** *M. mycetomatis* Grain size in *G. mellonella* larvae upon exposure to 332 µM EDDA, 25 µM FeCl_2_, and 25 µM FeCl_3_. Data are presented as mean values ± SD, determined based on five biological replicates. **E** Expression of *G. mellonella* genes related to iron regulation during infection upon exposure to 332 µM EDDA, 25 µM FeCl_2_, and 25 µM FeCl_3_ (*n* = 15, 3 biological replicates per time point). Data are presented as mean values ± SEM. We computed a two-way ANOVA test. **F** Expression of *M. mycetomatis* genes related to iron regulation during infection in *G. mellonella* larvae upon exposure to 332 µM EDDA, 25 µM FeCl_2_, and 25 µM FeCl_3_ (*n* = 15, 3 biological replicates per time point). Data are presented as mean values ± SEM. **G** Grocott stain of a *M. mycetomatis* grain in *G. mellonella* 72 h after infection and exposure to FeCl_3_. The arrows indicate the hyphae growing outside of the grain. The data shown are an example based on observations from five evaluated biological replicates. Source data for (**A**–**F**) are provided as a Source Data file.

When iron was restricted in *G. mellonella* using iron chelator ethylenediamine-N,N’-diacetic acid (EDDA), no difference in survival of healthy and *M. mycetomatis*-infected larvae was noted (Fig. 8A, B). However, when an iron overload was created with FeCl_2_, a trend towards lower survival was noted after infection with *M. mycetomatis* (Fig. 8B). Also, no significant changes were noted in the expression of iron-regulatory pathways in host (Fig. 8E, e.g., ferritin, transferrin or aconitase hydratase encoding genes). However, we did observe changes in the expression of the siderophore biosynthesis pathway. SidI, SidD, and SidA were differentially expressed under both iron-limiting and iron-overload conditions compared to the control (Fig. 8F). Under iron-overload conditions, grain formation seemed to be affected. Grain formation itself seemed delayed (Supplementary Figs. S9 and S10), and the number of grains was lower in FeCl_2_ and FeCl_3_-supplemented larvae compared to the control larvae (Fig. 8C, *P* = 0.0242 and *P* = 0.0019, respectively). Exposure to FeCl_3_ also resulted in a smaller total grain size (Fig. 8D, *P* = 0.0499) and less melanisation, especially early in the infection. Hyphae also grew easily out of the grains, a feature not reported before (Fig. 8G). Under iron-restricted conditions, melanisation became more intense at the periphery of the grain (Supplementary Figs. 9 and 10).

## Discussion

We demonstrated that several of the *G. mellonella* and *M. mycetomatis* genes are differentially expressed during grain formation. GSEA of differentially expressed genes reveals that most of the enriched GO terms of both host and pathogen are linked to energy pathways, nucleobase metabolic process, and cation and iron transport. Based on the numerous genes involved in iron homeostasis identified in the transcriptomics analysis, we focused on this process in both the host and pathogen.

To limit the access of iron for pathogens such as *A. fumigatus*, *Cryptococcus neoformans*, and *Mycobacterium tuberculosis*^30–32^ is an important innate defense mechanism from the host. This process, also known as nutritional immunity^33^, is most studied in mammals, little in insects, and hardly in *G. mellonella*. Therefore, our data adds to the knowledge of the iron response in *G. mellonella* during infection. Generally, in insects, iron sequestering, transport, and storage are regulated by both ferritin and transferrin^21,34,35^.

**Fig. 9:**
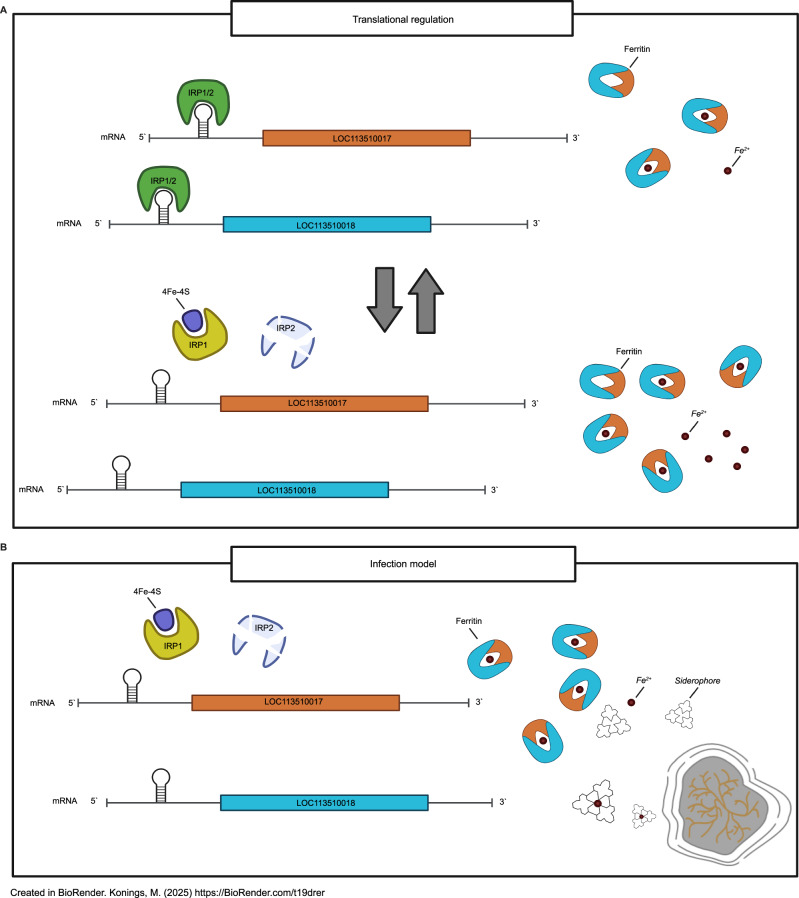
Proposed model of Iron regulation in *G. mellonella* during infection with *M. mycetomatis* “Created in BioRender. Konings, M. (2025) https://BioRender.com/t19drer”. **A** In healthy larvae, in the absence of iron (Fe^2+^), the IRP1 and IRP2 form a complex, which can bind to Iron-Responsive Elements (IRE) in the 5′ prime UTR region of the ferritin subunits coding transcripts LOC13510017 and LOC13510018, preventing translation of the mRNA to form ferritin. In the presence of iron (Fe^2+^), 4Fe-4S is formed, binding to IRP1. The IRP1/4Fe-4S complex prevents binding to the IRE, enabling translation of both LOC13510017 and LOC13510018, forming ferritin. Ferritin will bind Fe^2+^ for transportation and storage. **B** During infection, increased expression of aconitate hydratase results in increased formation of the 4Fe-4S complex, binding of IRP1, and thus the translation of both subunits of ferritin for the binding of Fe^2+^, creating an iron-derived environment. In response to the limited available iron, the fungus shows significantly increased expression of the siderophore-related biosynthesis pathway for the production of siderophores needed for sequestering Fe^2+^, which is essential for the survival of the fungus.

In our study, we did find expression of the ferritin subunit (LOC113510018), ferritin lower subunit (LOC113510017), and transferrin (LOC113509694) at all time points investigated. However, in contrast to what was seen in other fungal infections, expression levels did not significantly alter during infection^36^. Transferrin and ferritin protein levels previously measured in the same *G. mellonella* infection model, on the other hand, were differentially abundant. In that study, a 5- to 12-fold decrease in transferrin protein levels and a 3-fold increase in ferritin protein levels were noted during *M. mycetomatis* grain formation in *G. mellonella*^15^. For ferritin, this difference can be explained by the process of iron regulation via iron-regulatory proteins (IRP). In *G. mellonella*, these IRPs are encoded by aconitate hydratases, and *G. mellonella* aconitate hydratases LOC113516537, LOC113511518, and LOC113522652 were differentially expressed during *M. mycetomatis* infection. IRPs bind IREs under low iron conditions to repress the translation of ferritin. A process well studied in mammals^26,37^. In mammals, IRPs respond to low iron by binding to IREs present in the 5′UTR of the mRNAs of ferritin^33^. This results in translational repression of ferritin. In contrast, in the presence of enough iron, the enzyme aconitate hydratase will form a [4Fe-4S] cluster, which prevents the IRP from binding to the IRE. This, in turn, results in translational activation of ferritin^37^. Since we and others identified IREs in the 5′UTR regions of ferritin from *G. mellonella* and in *D. melanogaster*, and it was described that IRP1 was able to bind to the ferritin IRE to regulate translation, we postulate that in *G. mellonella*, the regulation of ferritin is similar (Fig. 9)^24–26^.

Due to the lack of IRE in the transferrin UTR regions, the discrepancy between transferrin expression levels and protein levels cannot be due to IRP/IRE regulation. Here, we showed that *M. mycetomatis* growth increased in vitro upon exposure to both apo- and holo-transferrin and ferritin, suggesting that *M. mycetomatis* is able to use these proteins as a nutrient source in a nutrient-limited environment. Furthermore, upon complete growth inhibition by exposure to the iron chelator 2′2-bipiridyl, the presence of iron-bound ferritin restored *M. mycetomatis* growth, suggesting that, in vitro, the fungus can obtain iron from iron-bound ferritin, but not from transferrin. In other pathogenic fungi, such as *Candida albicans* and *A. fumigatus*, it has been demonstrated that mammalian transferrin can be used as an iron source for the fungus^38,39^. *C. albicans* forms a complex with transferrin and obtains iron via the high-affinity reductive system^38^. In *A. fumigatus*, siderophores play a role in the removal of iron from transferrin^39^. The time needed for *A. fumigatus* to produce enough siderophores to remove iron from transferrin was 8 h, however, 22 h were needed to degrade the complete transferrin protein^39^. In our larval model, it took 24 h to get a 5-fold decrease in transferrin protein abundancy and 72 h to get a 12-fold decrease in transferrin protein abundancy in the mycetoma grain^15^. Furthermore, in the current study, we also demonstrated that in our *M. mycetomatis* larval infection model, the genes involved in the siderophore biosynthetic pathway were differentially expressed during infection. After 24 h of infection, a strong increase in the expression of especially SidA, SidD, and SidI was observed (Fig. 5), coinciding with the decrease in transferrin protein abundancy; this indicates that a similar system could exist in *M. mycetomatis* during infection.

Next to obtaining iron from transferrin, fungal siderophores can also obtain iron from other sources surrounding the fungus. Our PPI analysis demonstrated that almost all homologs of the *A. fumigatus* siderophore biosynthetic pathway were present in *M. mycetomatis*. Furthermore, LC-MS/MS data confirmed production of SidA and SidD by *M. mycetomatis* under iron-limited conditions. Only the homolog of the *A. fumigatus* gene encoding for FsC-acetyl coenzyme A-N(2)-transacetylase (SidG), the final enzyme required for the production of the *A. fumigatus* siderophore triacetylfusarinine C (TAFC), was not found in the *M. mycetomatis* genome. SidG is only required for the biosynthesis of TAFC, not for the siderophore fusarinine C (Fig. 5C), therefore, it is not surprising that there was no homolog in *M. mycetomatis*^40^. So far, homologs of SidG have only been found in a few *Aspergillus, Fusarium*, and *Nectria* species, but not in species lacking TAFC. Based on this, it is most likely that *M. mycetomatis* produces an extracellular fusarinine C-type siderophore and one or two intracellular ferrichrome-type siderophores, such as ferricrocin and hydroxyferricrocin. Here, we demonstrated the ability of *M. mycetomatis* to synthesize a hydroxamate-type siderophore under iron-limited conditions. This finding is in line with a previous finding that demonstrated the presence of hydroxamate-type siderophores, which are often composed of trans-fusarinine units, to be produced by *M. mycetomatis*, however, they were never chemically characterized^41^.

The exact role of the acquisition of iron via transferrin or siderophores in the pathogenicity of *M. mycetomatis* has not been described. However, in *A. fumigatus*, it has been demonstrated that the elimination of the entire siderophore biosynthesis resulted in avirulence in a murine model of invasive aspergillosis^42,43^. Furthermore, it has also been described that siderophores can be used either diagnostically or therapeutically^44^. Concurrent RP-HPLC analysis of culture supernatants identified the time-dependent secretion of 3 metabolites (P1–P3), however, extensive future work is required to determine the precise mass, stoichiometry, and structure of the ferrated siderophore (present in P2).

In this study, we used a transcriptomic approach to decipher the response of the *G. mellonella* host and the *M. mycetomatis* pathogen in eumycetoma grain formation. Although a large number of genes were differentially expressed, we demonstrated that iron metabolism plays an important role in *M. mycetomatis* grain formation in *G. mellonella* over time during infection. A limitation of this model is that we could only study the innate immune response; the adaptive immune response present in mammals but absent in insects could not be studied. Despite the absence of the adaptive immune response, grains were still formed in this model, and we found a large number of genes differentially expressed. We also demonstrated that iron metabolism plays an important role in *M. mycetomatis* grain formation in *G. mellonella* over time during infection.

The data generated in this study can be reused to answer other biological questions, including research in drug development (enhance the current limitation in eumycetoma treatment) and study on mycoviruses in *M. mycetomatis*. The dataset can be used to enhance the current annotation of the host *G. mellonella* and *M. mycetomatis* genomes. The identification of the importance of iron acquisition during grain formation can be exploited as a potential novel diagnostic and therapeutic strategy for mycetoma.

## Methods

### Culturing and inoculum preparation of *M. mycetomatis* strain MM55

Preparation of the inoculum was performed as previously described by Kloezen et al.^8^. In short, *M. mycetomatis* genome strain MM55 was cultured on Sabouraud Dextrose Agar for 2–3 weeks at 37 °C. Mycelium was harvested, sonicated at 28 micron (Soniprep 150, Beun de Ronde, The Netherlands) and incubated at 37 °C in RPMI-media supplemented with 0.35 g/L L-glutamine, 1.98 mM 4-morpholinepropanesulfonic acid (MOPS) and 100 mg/L chloramphenicol. After 2 weeks, the mycelia were separated and washed by vacuum filtration (Nalgene, Abcoude, The Netherlands using a 22 micron filter (Whatman)). The mycelial biomass was scraped from the filter, the wet weight was determined, and a suspension was prepared containing 100 mg mycelial biomass per ml phosphate-buffered saline (PBS). The suspension was sonicated at 28 micron for 2 min (Soniprep 150, Beun de Ronde, The Netherlands), the resulting homogeneous suspension was washed once in PBS and again diluted to a concentration of 4 mg per 40 µL PBS, corresponding to 600–850 CFU/larvae.

### Infection of *G. mellonella* larvae with *M. mycetomatis* and RNA isolation

*G. mellonella* larvae were obtained from Terra Equipment Voedseldieren (Cuijk, The Netherlands) and kept in the dark on wood shavings at room temperature until use. Within 5 days of receipt, larvae of ~300–500 mg were selected for experimental use. The selected larvae were divided over Petri dishes containing 90 mm Whatman filter paper, and five larvae per dish. Forty microliters of the prepared inoculum of *M. mycetomatis* strain MM55 was injected in the last left proleg of the larvae using an insulin 29G U-100 needle (BD diagnostics, Sparks, USA), resulting in a final concentration of 4 mg fungal biomass/mL. To monitor the course of the infection, a separate group consisting of 15 larvae was infected, and survival was recorded daily for the duration of 10 days. During all experiments, Pupa were removed from the equation, and non-infected larvae were included as controls. At 4, 24, 72, and 168 h post-infection, the contents of three larvae were pooled and flash-frozen with liquid nitrogen, followed by mechanical crushing using a pestle and mortar. The resulting powder was suspended in RLT buffer (supplemented with 1% β-Mercaptoethanol), provided in the RNeasy Mini Kit (Qiagen, Germany), and incubated at 57 °C for 3 min. RNA isolation was continued according to the manufacturer’s instructions. The presence and quality of RNA were assessed by NanoDrop and gel electrophoresis.

### Determination of the fungal burden in *G. mellonella*

At 4, 24, 72, and 168 h post-infection, larvae were sacrificed, and haemolymph was harvested. Melanisation of haemolymph was determined by diluting the haemolymph 1:1 in (IPS). The optical density was measured at 405 nm using the Nanodrop (Thermo Scientific, USA). To fixate the larvae, 100 µL of 10% buffered formalin was injected into the larvae. The larvae were moved into 15 mL Greiner tubes containing 5 mL 10% buffered formalin. After 24 h of fixation, whole larvae were longitudinally dissected into two halves using a scalpel and fixated in 10% buffered formalin for another 48 h before further routine histological processing^45^. The two halves were stained with hematoxylin and eosin (H&E) and Grocott methenamine silver for further histological examination. The fungal burden was determined by manually counting the grains using 40× magnification of a light microscope mounted with a Canon EOS70D camera (Canon Inc.). Counting was performed by three independent scientists. The grains were visualized on a computer screen using EOS Utility software (Canon Inc.) and categorized into large (>0.02 mm^2^), medium (0.01–0.019 mm^2^), and small (0.005–0.009 mm^2^) sizes as described by Lim et al.^46^. The sum of all grains represents the total amount of grains within the larvae. The total grain size within the larvae was determined by multiplying the sum of all grains with the minimum size of their respective categories. The difference in the total number of grains or the total grain size observed on the respective time points was determined using the Mann–Whitney *U* test in GraphPad Prism 8. A *p* value > 0.05 was deemed significant.

### RNA-Seq and LQ-ssCAGE library preparation, sequencing, mapping, and processing

Total RNA undergoes QC using NanoDrop and Bioanalyzer. Required RNA QC for library preparation was obtained (Supplementary Note 1).

As illustrated in Fig. 2A, we prepared two types of libraries for high-throughput profiling, RNA-Seq and LQ-ssCAGE libraries (See Supplementary Notes 2–4 for LQ-ssCAGE). A full list of samples is shown in Supplementary Data 1. RNA-Seq libraries were prepared using 1 μg of total RNA with the TruSeq Stranded mRNA Library Prep kit (Illumina) following the manufacturer’s instructions. Transcriptomics profiling was performed on a distinct sample.

HiSeq2500 instrument from Illumina was used for sequencing with Paired-End, 100 base, followed by base calling. The quality of the raw reads was assessed using FastQC [https://www.bioinformatics.babraham.ac.uk/projects/fastqc/]. We returned 737,882,376 total tags for the *G. mellonella* larvae samples and 152,647,712 total tags for the *M. mycetomatis* samples (Supplementary Note 1).

*M. mycetomatis* raw reads mapped against *M. mycetomatis* genome assembly ASM127576v2^47^. The *G. mellonella* larvae reads were mapped twice, first against the *G. mellonella* genome assembly ASM364042v2 and then against the *M. mycetomatis* genome assembly ASM127576v2. We used STAR Aligner version 2.7.1a with default settings^48^. In short, for each of the *M. mycetomatis* libraries, on average, 43.8 million reads were mapped, of which, on average, 75.8% were uniquely mapped. For each of the *G. mellonella* larvae libraries, on average, 49.1 million reads were mapped. In the *G. mellonella* larvae libraries, 68,398 reads mapped to the *M. mycetomatis* genome assembly ASM127576v2.

RNA-Seq runs for the study of the impact of iron on the growth of the fungal and infection on the host (Fig. 1A, right), RNA-Seq libraries (*n* = 36) prepared using NEBNext Ultra II RNA Library Prep. All libraries (*n* = 36) passed the library QC and were submitted for sequencing (Supplementary Note 4). Paired-End sequence with NextSeq 2000 was successfully completed. We applied the same workflow as above for base calling, mapping of the raw sequence reads to the host and pathogen genomes, and read summarization. We obtained 1,293,061,642 total number of reads in this run. Applying UMAP clustering shows perfect clustering of the sample per time point (0, 4, 24, and 72 h) (Supplementary Note 4). To quantify the mapped reads from RNA-Seq libraries, the Rsubread FeatureCount function was utilized using the same parameters for RNA-Seq quantification^49^.

### Exploratory analysis of RNA-Seq and LQ-ssCAGE data

The previously generated raw count matrix for RNA-Seq and LQ-ssCAGE was used for exploratory data analysis to explore the clustering of the samples per time point and expression patterns using the R implementation of the t-distributed stochastic neighbor embedding (*t-SNE*). The Rtsne function of the Rtsne package (V 0.15) with is_distance = TRUE and perplexity = 5. To explore the clustering of the host samples from the LQ-ssCAGE dataset mapped to the host genome, the prcomp function from stats (V 3.6.2) was used with standard settings and perform principal component analysis.

### Differential expression analysis

Raw RNA-Seq counts were normalized using the edgeR R data package^50^. First, we generated the DGElist object and then calculated the normalization factor for the raw reads using the calcNormFactors function and trimmed the mean of the M value. The recommended count per million (CPM) was obtained as a normalized gene expression matrix. The normalized expression values were used for downstream analysis to identify differentially expressed genes and perform advanced computational analysis of enriched pathways.

In order to process time course data and understand the overtime changes in the eumycetoma grain in the infected larvae, a differential gene expression analysis workflow was designed (Supplementary Fig. 11). This workflow was used to analyze two types of changes. First, the sharp change analysis, which detects the changes between pre-infection and post-infection (e.g., T0 and T168). The sharp changes were detected using a two-step regression model analysis implemented in the maSigPro R package^51^. The result of this analysis is depicted in (Fig. 3A). The second type of change was the consecutive change, which was detected by computing the changes between consecutive time points and between every time point and T0. To detect consecutive changes, a linear model analysis (LIMMA) embedded in the edgeR R package was used.

To identify common DGE between consecutive and sharp changes, the results from the two steps of regression analysis and the linear model analysis were further integrated. In short, a final LIMMA-derived *P* value was computed for each gene by combining all contrasts (e.g., T0–T4h) using the eBayes function of the LIMMA R package and computing the *F*-statistic, the associated *P* value, and the adj. *P* value using Benjamini–Hochberg multiple testing correction. A gene was differentially expressed if any of the following conditions applied: (a) LIMMA FDR < 0.001, (b) maSigPro *R*^2^ > 80%, or (c) LIMMA FDR < 0.01 and maSigPro *R*^2^ > 60%.

Results from the Differential gene expression (DGE) analysis were visualized using a standard heatmap with customized settings for the heatmap.2 functions from the R package gplots (V 3.1.1). In addition to the heatmap, the EnhancedVolcano R package was used to plot the Volcano plots and visualize the results from the DGE.

### Detect the pattern of expression from DGE

To understand the expression pattern over time, the DEG from LIMMA and maSigPro of the RNA-Seq and LQ-ssCAGE data was analyzed. This kind of analysis shows the dynamic gene expression per time point and the host response to the infection. The function degPatterns from the DEGreport R package (V 1.26.0) was used to cluster genes with similar expression patterns over time. The input for the degPatterns function expression matrix in logarithmic scale (only genes that are significantly different), the time course design experiment used to group samples (Healthy vs. infected larvae). In the degPatterns, the following parameters were set: The minc (minimum number of genes in a group that will be returned) = 15; The parameter summarize we set to “merge”; The results of the DEG patterns for *G. mellonella* larvae RNA-Seq reads mapped to host genome (Supplementary Fig. 12); and the results of the DEG patterns for *G. mellonella* larvae RNA-Seq reads mapped to pathogen genome (Supplementary Fig. 13). Similarly, the DEG TSS patterns were analyzed for *G. mellonella* larvae reads mapped to the host genome (Supplementary Fig. 14). The detected gene pattern of the DEG from RNA-Seq is further summarized and grouped in Fig. 4.

### Gene set enrichment and pathway analysis

Gene set enrichment analysis for the host and pathogen was performed using the clusterProfiler R package (V 3.0.4)^52^. The function gseGO of clusterProfiler requires an input of the ordered rank gene list from edgeR, ordered by logFC. The DEG list from edgeR was annotated with the Gene Ontology terms from Uniprot^18^. In addition to the annotated gene list, gseGO requires the AnnotationDbi orgDB package. Since the orgDB was for the *G. mellonella* and *M. mycetomatis* in R Bioconductor, we created two new orgDBs for each species. We ran gseGO for each of the gene ontology categories (CC, BP, and MF). The number of permutations was specified as 1000, and the *p* value cutoff was defined as “=0.05.” For each of the contrasting time points, the result of the functional enrichment analysis was visualized using a dotplot and emapplot. *P* adjusted value for each plot was shown as well.

### Identification and analysis of 1,8-Dihydroxynaphthalene (DHN)-melanin, pyomelanin, and siderophore biosynthesis pathways in *M. mycetomatis*

We investigated iron regulation in *G. mellonella* in three biosynthesis pathways in *M. mycetomatis*. The three pathways are the 1,8-Dihydroxynaphthalene (DHN)-melanin, pyomelanin, and siderophore. Since these pathways are not yet characterized in *M*. *mycetomatis*, we used the fungal * A.  fumigatus* [NCBI: taxid746128] as a surrogate species (proxy) to identify the genes involved in the regulation of these pathways. *A. fumigatus* genes involved in DHN-melanin (6 genes) and pyomelanin (6 genes) were fetched from T. Heinekamp et al.^53^. And for the siderophore biosynthesis pathway, the eight genes were fetched from Gründlinger et al.^28^. After collecting the set of genes involved in each biosynthesis pathway in *A. fumigatus*, we looked at their homologous genes in *M. mycetomatis* (Supplementary Note 5). After obtaining *A. fumigatus* homologous in homologous genes in *M. mycetomatis* from BLAST^20^, we used the protein-protein interaction database STRING to identify possible functional interactions between the genes for each pathway^54^ (Supplementary Note 5).

### Validation of selected *G. mellonella* and *M. mycetomatis* gene expression quantitation by Real-Time Quantitative Reverse Transcription PCR (RT-qPCR)

Validation by RT-qPCR of the expression quantitation of a selected set of differentially expressed genes of the *G. mellonella* and *M. mycetomatis* was performed. The cDNA was synthesized from 1 μg of total RNA with the oligo (dT)20 primer and SuperScript III Reverse Transcriptase (ThermoFisher) following the manufacturer’s instructions. RT-qPCR was performed using synthetic primers (Supplementary Data 2), TaKaRa Ex Taq HS (Takara Bio), and SYBR Green I Nucleic Acid Gel Stain (ThermoFisher). For each gene in (Supplementary Data2), a DNA fasta file was retrieved from the NCBI nucleotide database. The DNA fasta file, used as input for Primer3Plus^55^ to pick primers from a DNA sequence. The selected primers by Primer3Plus were further processed with Multiple Primer Analyzer (ThermoFisher) and with The Sequence Manipulation Suite^56^. PCR was performed for 35 cycles under the following conditions: 98 °C for 10 s, 56 °C or 64 °C for 30 s, and 72 °C for 60 s.

### *M. mycetomatis* iron-deplete cultures

Iron-deplete media pH 6.5;10 g of D-glucose and 1.9 g of Formedium CYN1201 (Yeast Nitrogen Base without amino acids, ammonium sulfate, and iron) were dissolved in 950 mL of deionized water. pH was adjusted to 6.5 using 1 M HCl and 1 M NaOH, and the volume was adjusted to 1000 mL. The solution was autoclaved at 115 °C for 30 min. After the solution cooled to room temperature, 66 mL of sterile 0.3 M L-glutamine in deionized water was added to the solution. Next, *M. mycetomatis* genome strain mm55 was grown on Sabouraud Dextrose Agar for 2–3 weeks at 37 °C. Mycelia were extracted from the agar using a sterile inoculating loop and used to inoculate 20 mL of iron-deplete media, pH 6.5, in triplicate. Cultures were incubated at 37 °C and 170 rpm for 7 days. One milliliter aliquots were taken from the cultures at 0, 24, 48, 72, 96, and 168 h. After 168 h, the mycelia were extracted, dried, and snap frozen in liquid nitrogen and stored at −20 °C. Remaining culture fluid was stored at 4 °C.

### Proteomic analysis of *M. mycetomatis* mycelia

Dried *M. mycetomatis* mycelia were ground in liquid N_2_ using a mortar and pestle. 300 mg of ground mycelia was resuspended in 1 mL of FASP lysis buffer (250 mg of sodium dodecyl sulfate, 250 µL of 0.1 mM PMSF and 10 µL of 10 mg/mL leupeptin in 25 ml 50 mM ammonium bicarbonate pH 7.8.) and homogenized by vortexing and sonicating four times at cycle 6 at 20% power for 30 sec. Protein concentration was quantified by Qubit. 20 µg of protein was prepared for LC-MS/MS analysis as previously described^57,58^ and detailed in (Supplementary Note 6). Following isolation, LC-MS/MS analysis of mycelial digests was performed as follows 250 ng of tryptic peptides from each sample was then analyzed by a Thermo Fisher Q-Exactive mass spectrometer coupled with a Dionex RSLCnano. LC gradients ran from 3 to 40% solvent B over 2 h and 13 min, and data were collected using a Top 15 method for MS/MS scans. Analysis of peptides was performed by Proteome Discover (ver. 1.4.0.288) with a medium peptide confidence and a minimum of two peptides per protein as filters. Protein amino acid sequence homology was performed by BLAST^20^.

### Analysis of *M. mycetomatis* culture supernatants

Both RP-HPLC and LC-MS analysis was performed to analyze culture supernatants as previously described^59,60^. For RP-HPLC, 1 µL of 1 M FeSO4 was added to culture media collected at the indicated time points, mixed, incubated at 37 °C for 30 min, and centrifuged at 14,000 × *g* for 10 min. 20 µL of the ferrated supernatants were then analyzed by an Agilent 1200 series RP-HPLC system with a diode array detector (DAD) with a C8 analytical column (Agilent Zorbax Eclipse XDB-C8 Analytical; 5 μm particle size; 4.6 × 150 mm) used for separation of analytes with DAD detection at 230, 254, 330 and 440 nm. Solvent A: 1 mL of trifluoroacetic acid in 999 mL of deionized water. Solvent B: 1 mL of trifluoroacetic acid in 999 mL of acetonitrile. LC gradients ran from 5-100% solvent B over 40 min. For the LC-MS analysis, *M. mycetomatis* was incubated at 37 °C in iron-free media (50 ml each) in triplicate, and 170 rpm for 5 days before mycelia was extracted from the cultures, dried, and snap frozen in liquid nitrogen. Two 1 mL aliquots were taken from each culture supernatant with 1 µL of 1 M FeSO4 added to one of the aliquots from each supernatant, which were then mixed by vortexing, incubated at 37 °C for 30 min, and centrifuged at 14,000 × *g* for 10 min. Twenty microliters of supernatants with and without iron were analyzed by RP-HPLC^59,60^, and fractions containing siderophore were collected and dried at 37 °C. The remaining culture supernatants were snap frozen and stored at −20 °C. For LC-MS/MS analysis, dried samples were resuspended in LC-MS/MS loading buffer, sonicated for 2 min, and centrifuged at 14,000 × *g* for 10 min. Resuspended samples were analyzed by a Thermo Fisher Ascend mass spectrometer coupled with a NeoVanquish LC system. LC gradients ran from 3 to 90% solvent B over 30 min with a *m/z* range of 350–2000. Analysis was performed on Thermo Freestyle.

### Fungal growth under iron supplementation

*M. mycetomatis* genome strain MM55 was cultured on Sabouraud Dextrose Agar for 2 weeks at 37 °C. Disks of 2 mm were punched out of *M. mycetomatis* mycelium and transferred to Yeast Nitrogen base without Amino Acids and without Iron (YNAAI) agar plates supplemented with 25 µM of FeCl_2_, FeCl_3_, apo-transferrin, holo-transferrin, apo-ferritin, and holoferritin. PBS was included as a control, and these conditions were also replicated under exposure to an inhibitory concentration of 500 µM of the iron chelating agent 2,2′-bipyridyl. After 0, 7, and 14 days of growth at 37 °C, the diameter of the mycelium was measured. Two biological replicates were performed (Fig. 1A, middle).

### Survival of *G. mellonella* exposed to different iron-influencing conditions

*G. mellonella* larvae were injected with FeCl_2_ and FeCl_3_ to a final concentration of 25 µM to create an iron overload, and 332 µM ethylenediamine-N,N’-diacetic acid (EDDA) based on previous findings of Dunphy et al. in order to create an iron defficit^61^. Healthy larvae were first used to determine the toxicity of the different conditions to the larvae. Next, larvae were injected with either FeCl_2_, FeCl_3_, or EDDA to final concentrations as stated above. After 30 min, the larvae were infected with *M. mycetomatis* strain MM55, and survival was monitored for 10 days. Additionally, after 4, 24, 72, and 168 h, larvae were sacrificed for RNA isolation and histological examination as described above (Fig. 1A, right).

### Statistics and reproducibility

To assess the burden of infection in the *G. mellonella*, we used GraphPad Prism version 8.4.3 (GraphPad Software, LLC). For the rest of the analysis, we used R version 4.1.2 (2021-11-01). Data are represented as either box-and-whisker plots, line plots, or bar plots (represented as expression ± SEM) as specified in the figure legends. For testing differentially expressed genes, the *F*-statistic, the associated *P* value, and the adj. *P* value were corrected using Benjamini–Hochberg multiple testing correction.

### Reporting summary

Further information on research design is available in the Nature Portfolio Reporting Summary linked to this article.

## Supplementary information

Supplementary Information

Description of Additional Supplementary Files

Supplementary Data 1

Supplementary Data 2

Supplementary Data 3

Supplementary Data 4

Supplementary Data 5

Supplementary Data 6

Supplementary Data 7

Supplementary Data 8

Supplementary Data 9

Supplementary Data 10

Reporting Summary

Transparent Peer Review file

## Source data

Source Data

## Data Availability

Raw sequence data and expression tables used in the analysis have been deposited in the Gene Expression Omnibus (GEO) repository, accession numbers: GSE213321, GSE213322, GSE213329, GSE213332, GSE280443. The mass spectrometry proteomics data have been deposited in the ProteomeXchange Consortium via the PRIDE partner repository with accession number PXD063449. Source data are provided with this paper.

## Code availability

The two annotation hubs database used in GSEA, the R code files (. R), and data objects (RData) used to generate figures are available from https://dmsgrdm.riken.jp:5000/utk5m/.
