# Supplementary material for: Iron regulatory pathways differentially expressed during Madurella mycetomatis grain development in Galleria mellonella
Source: Nat Commun. 2025 Jun 25;16:5324. doi: 10.1038/s41467-025-60875-2 (PMC12198395; doi:10.1038/s41467-025-60875-2)
Supplement: Supplementary file 1 — Supplementary Information [file 41467_2025_60875_MOESM1_ESM.pdf]

Supplementary Information for

Iron regulatory pathways differentially expressed during *Madurella mycetomatis* grain development in *Galleria mellonella*

Imad Abugessaisa[1,2,3,¶,§,✉], Mickey Konings[4,¶], Ri-Ichiroh Manabe[5], Cathal M. Murphy[6], Tsugumi Kawashima[5], Akira Hasegawa[1], Chitose Takahashi[5], Michihira Tagami[5], Yasushi Okazaki[5], Kimberly Eadie[4], Wilson Lim[4], Sean Doyle[6], Annelies Verbon[4,7], Ahmed H. Fahal[8], Takeya Kasukawa[1], Wendy W.J. van de Sande[4, §,✉]

[1]Laboratory for Large-Scale Biomedical Data Technology, RIKEN Center for Integrative Medical Sciences, 1-7-22 Suehiro-cho, Tsurumi-ku, Yokohama City, Kanagawa, 230-0045, Japan.

[2]Premium Research Institute for Human Metaverse Medicine (WPI-PRIMe), The University of Osaka, 2-2, Yamadaoka, Suita, Osaka, 565-0871, Japan

[3]Graduate School of Medicine and Faculty of Medicine, The University of Osaka, 2-2, Yamadaoka, Suita, Osaka, 565-0871, Japan

[4]Department of Medical Microbiology and Infectious Diseases, Erasmus MC, University Medical Center Rotterdam, Dr. Molewaterplein 40, 3015GD Rotterdam, The Netherlands.

[5]Laboratory for Comprehensive Genomic Analysis, RIKEN Center for Integrative Medical Sciences, 1-7-22 Suehiro-cho, Tsurumi-ku, Yokohama City, Kanagawa, 230-0045, Japan

[6]Department of Biology, Maynooth University, Maynooth, Co. Kildare, W23 F2H6, Ireland.

[7]Department of Internal medicine, University Medical Center Utrecht, Heidelberglaan 100, 3584 CX Utrecht, The Netherlands.

[8]Mycetoma Research Center, University of Khartoum, Khartoum, Sudan.

[¶] These authors contributed equally

[§] These authors jointly supervised this work

[✉]e-mail: imad.abugessaisa@a.riken.jp ; w.vandesande@erasmusmc.nl

Table of contents

Supplementary figures 4

Supplementary Fig. 1 4

Supplementary Fig. 2 4

Supplementary Fig. 3 5

Supplementary Fig. 4 6

Supplementary Fig. 5 7

Supplementary Fig. 6 8

Supplementary Fig. 7 8

Supplementary Fig. 8 9

Supplementary Fig. 9 10

Supplementary Fig. 10 11

Supplementary Fig. 11 11

Supplementary Fig. 12 12

Supplementary Fig. 13 12

Supplementary Fig. 14 13

Supplementary Note [1] : Total RNA QC and total tag count (Run I) 13

1.1 NanoDrop QC 13

1.2 Bioanalyzer RIN value 14

1.3 Total tag count of the RNA-seq reads 14

Supplementary Note [2] : LQ-ssCAGE 15

2.1 LQ-ssCAGE library preparation 15

2.2 LQ-ssCAGE library sequencing 15

2.3 LQ-ssCAGE library mapping 17

2.4 LQ-ssCAGE TSS peaks calling 18

2.5 LQ-ssCAGE Prediction of the transcription factors binding sites (TFBS) and motif activity overtime 18

Supplementary Note [3] : G. mellonella promoters and active enhancers expression landscape during infection 19

3.1 TSS and enhancers 19

3.2 differential expression of the host TSS 20

3.3 TFBS prediction and motif analysis 20

Supplementary Note [4] : Total RNA QC and total tag count (Run II) 22

4.1 RNA quantification and library DNA 22
4.2 Total tag count and mapping rate 23
4.3 UMAP clustering of the samples 24
Supplementary Note [5] : Obtaining *A. fumigatus* homologous genes in *M. mycetomatis* 25
5.1 BLAST 25
5.2 Protein–Protein-Interaction network 25
Supplementary Note [6] : Proteomic analysis of *M. mycetomatis* mycelia 25
6.1protein preparation for LC-MS/MS analysis 26
Supplementary References 26

Supplementary figures

Supplementary Fig. 1

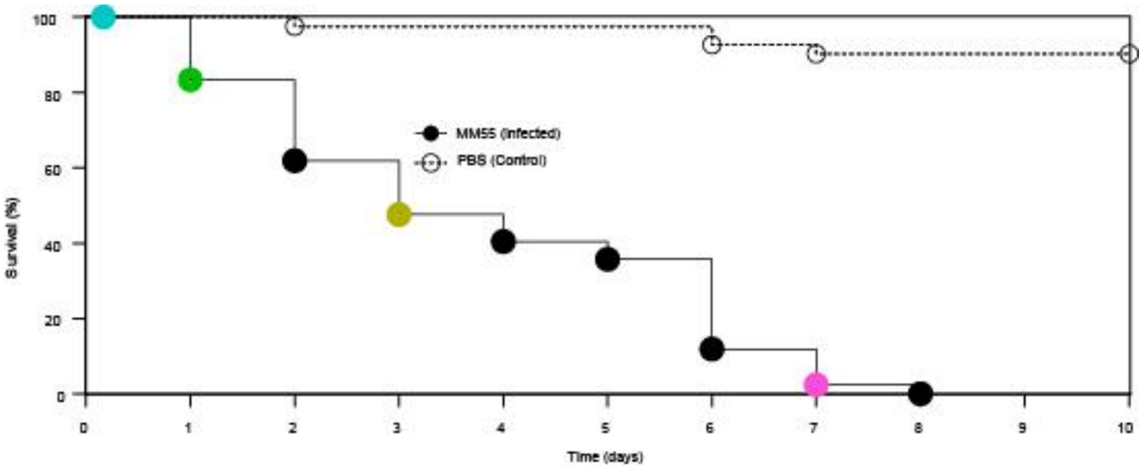

Supplementary Fig. 1: Larval survival curve. 15 larvae were infected and monitored for 10 days. All larvae died within 192 hours after infection. Source data are provided as a Source Data file.

Supplementary Fig. 2

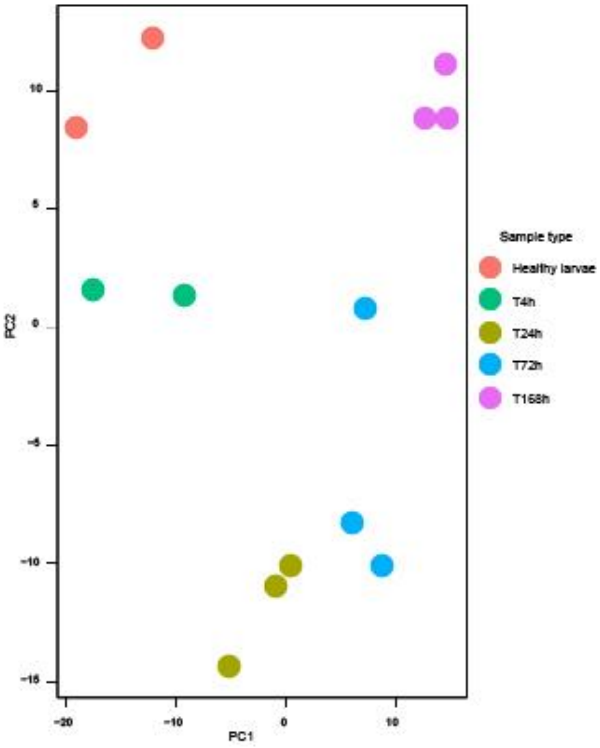

Supplementary Fig. 2: Clustering of LQ-ssCAGE samples, PCA: LQ-ssCAGE TSSs of host samples. (n=3), one sample excluded at time 0h and another sample excluded at time 72h. Source data are available in the Gene Expression Omnibus (GEO) under accession number GSE213332.

Supplementary Fig. 3

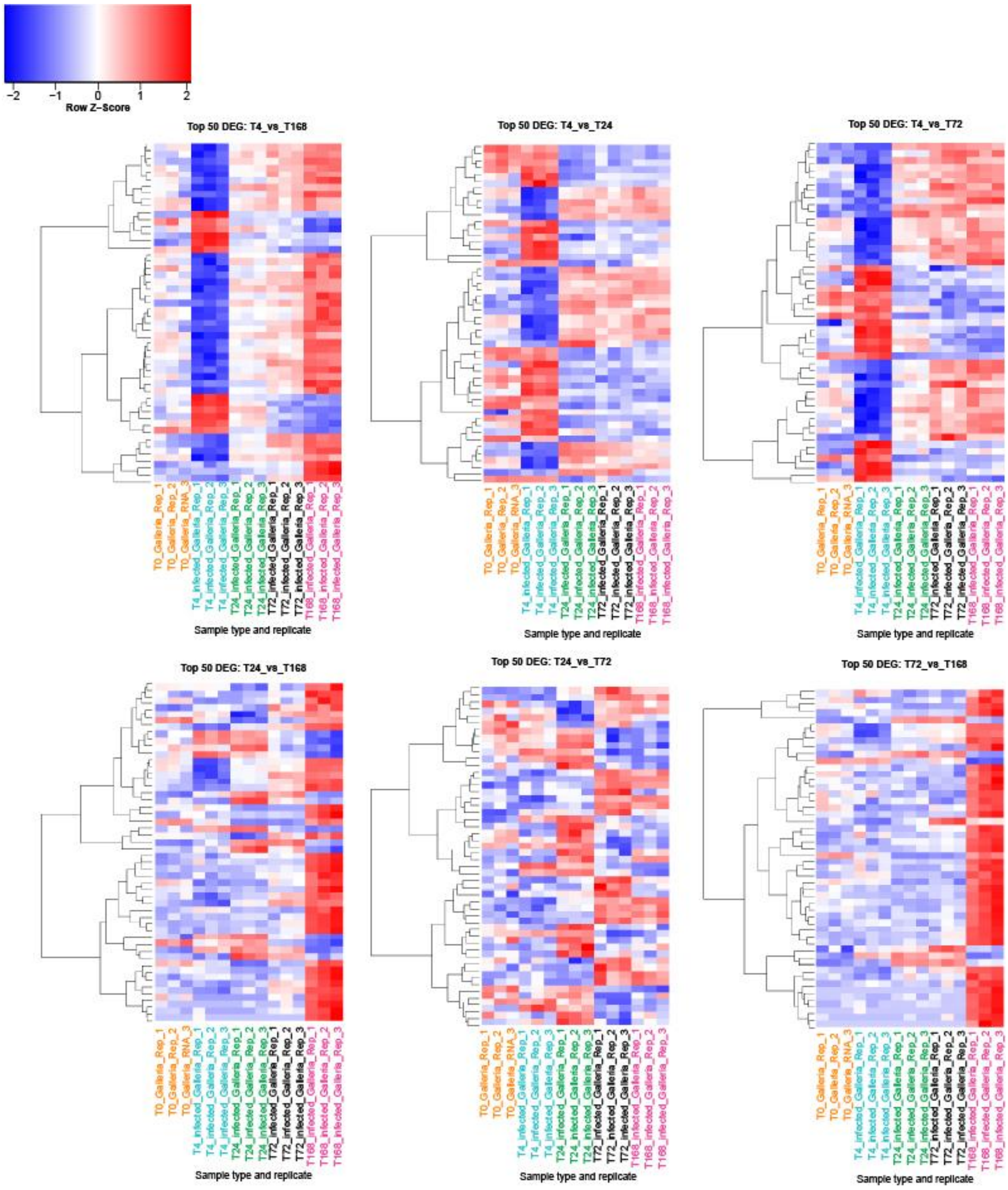

Supplementary Fig. 3: Heatmap of the top 50 host DEG from RNA-seq. 3 biological replicates per time point total number of samples (n=15). Source data are available in the Gene Expression Omnibus (GEO) under accession number GSE213329.

Supplementary Fig. 4

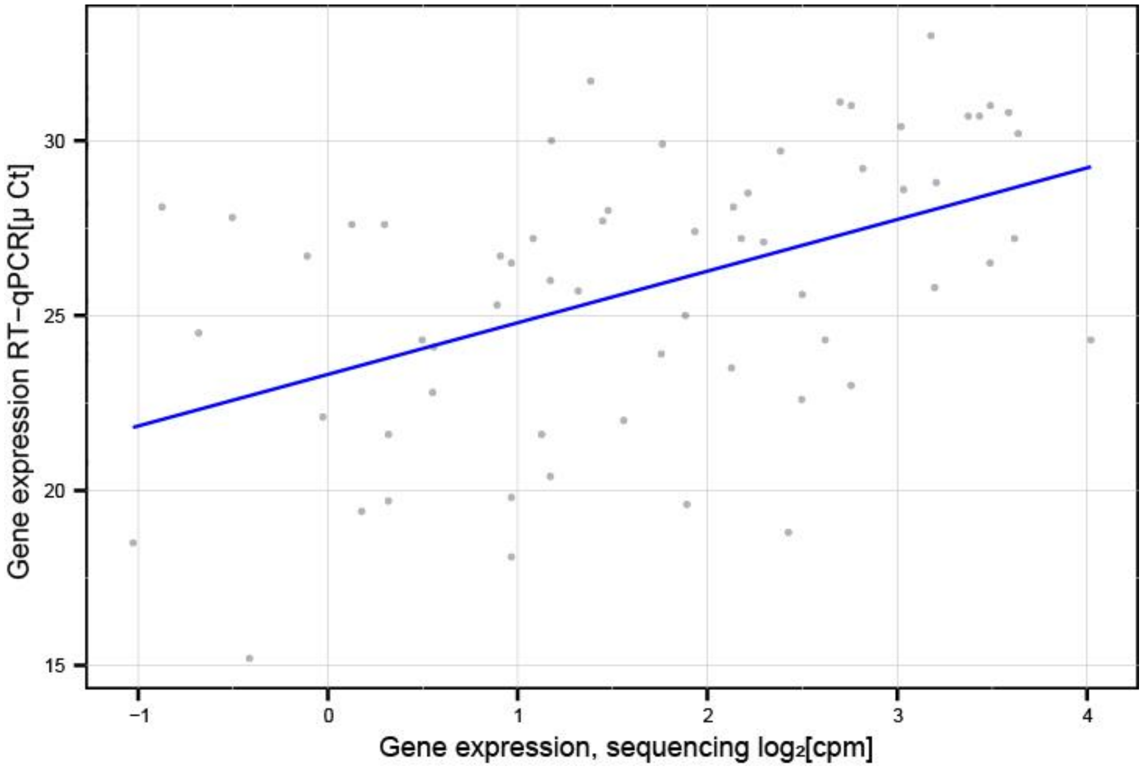

Supplementary Fig. 4: Validation of gene expression quantitation by RT-qPCR. Source data are provided as a Source Data file.

Supplementary Fig. 5

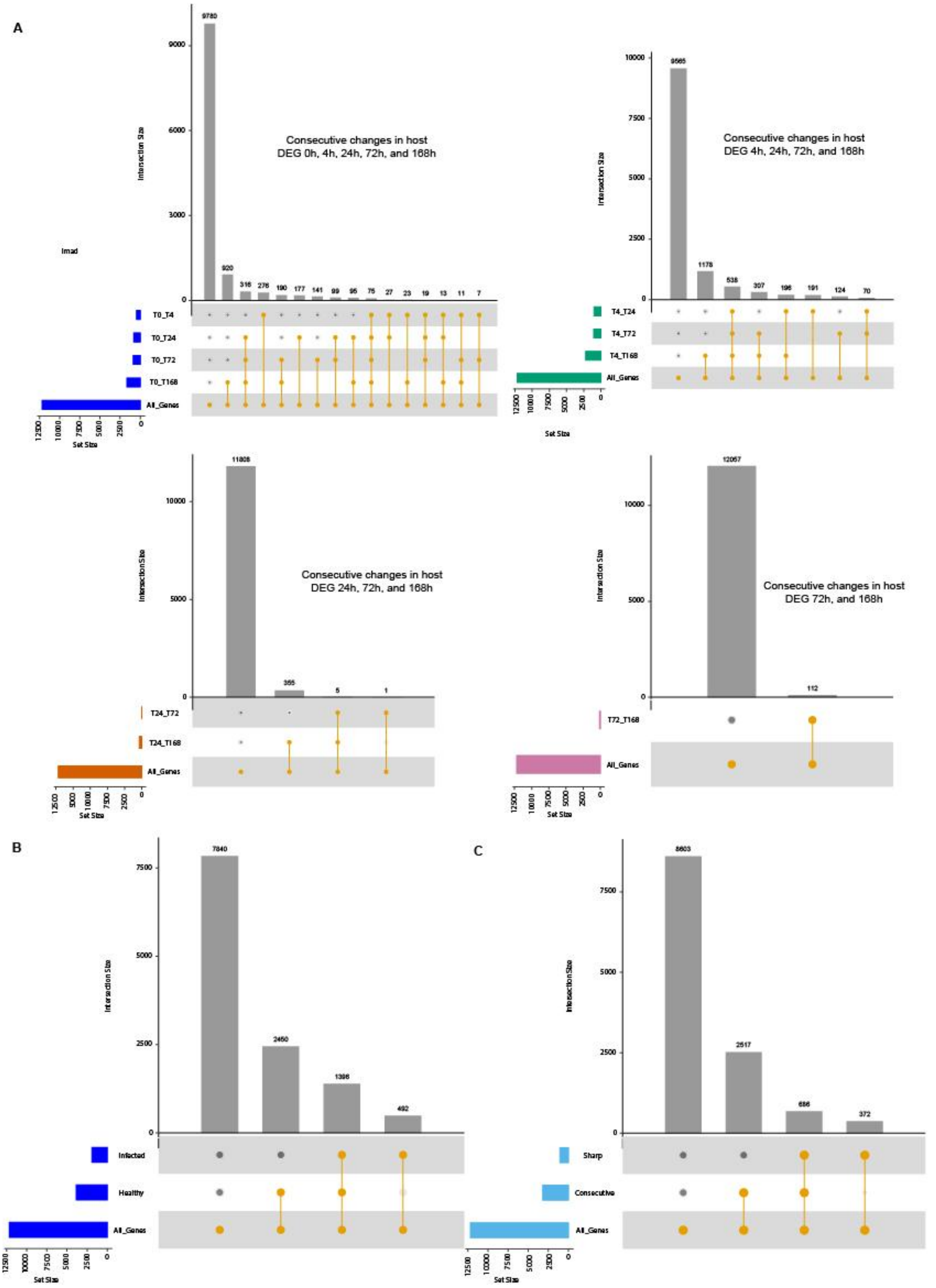

Supplementary Fig. 5: Upset plot of the summary of the consecutive changes in DEG. **A**. Consecutive changes in the host. **B**. Sharp changes in the host DEG Infected vs. Healthy. **C**. Common DEG between Consecutive and sharp changes in the host. Source

data are available in the Gene Expression Omnibus (GEO) under accession number GSE213329.

Supplementary Fig. 6

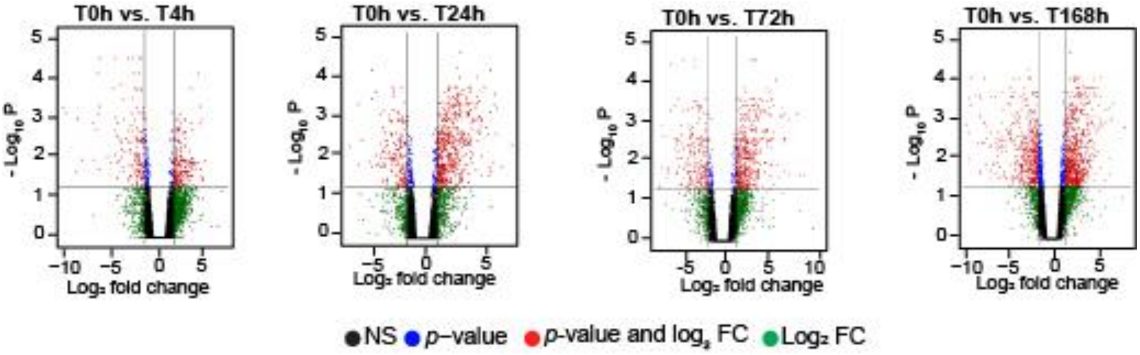

Supplementary Fig. 6: Volcano plot differential expression of the host RNA-seq. Source data are available in the Gene Expression Omnibus (GEO) under accession number GSE213329.

Supplementary Fig. 7

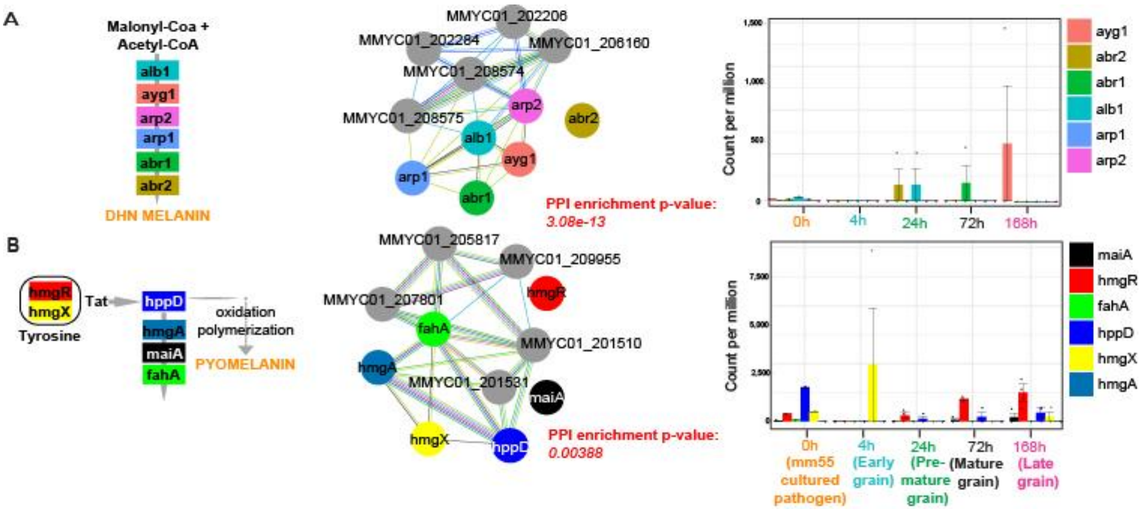

Supplementary Fig. 7: Biosynthesis pathways in *A. fumigatus* and homologous in *M. mycetomatis*. **A**. 1,8-Dihydroxynaphthalene (DHN)-Melanin biosynthesis pathway as defined in [1] (left), the PPI of the 1,8-Dihydroxynaphthalene (DHN)-Melanin biosynthesis pathway homologous in *M. mycetomatis* (middle), gene expression of the 1,8-Dihydroxynaphthalene (DHN)-Melanin biosynthesis pathway homologous in *M. mycetomatis* (right). **B**. Pyomelanin Biosynthesis pathways in *A. fumigatus* as defined in [1] (left), the PPI of the pyomelanin biosynthesis pathway homologous in M. mycetomatis (middle), gene expression of the pyomelanin biosynthesis pathway homologous in *M. mycetomatis* (right). In the bar chart, bar represents the mean value. 3 biological replicates per time points (n=15). Source data are provided as a Source Data file.

Supplementary Fig. 8

A. MSPHSLDDMD HPTANGTHSG DAVPRLVNGV SNGATGHGFP KSRFLRAADA DSVHDLVCVG FGPASLAIAV ALHDSLEAGK
LAQPPKVLFL EKQSQFAWHA GMLLPGARMQ ISFIKDLASL RDPRSHFTFL NYLHKNDR**LV DFTNLSTFLP ARVEYEDYLR**
WCARHFDDVV R**YQSEVLSVS PVLEEGPLK**T FTVASRNVKT EATSTYRAR**N VIVAVGGQAS IPRLFPEGHP R**VIHSSQYAQ
LVPKILADRS ATYR**VAIIGA GQSAAEIFSN VQTLYPNSR**T YMVMRSEFLK PSDDSPFVNS IFNPEFIDTL YPKSSTYRAN
LLHDARATNY GVVRLELIEH LYEIMYHQKR TLGSDEKQWP HRILAAR**DVI NVEEKGDKLH IK**VARTPVLG GPDGPLEEEG
LDVDLVICAT GYKRTAHVDM LKDTWHMLPE IDVAGKESGI PRKDR**WLVEA TNK**AHRGSST RVMEVGRDYG VRFSAGTVAP
GSGVWLQGCC EATHGLSDTL LSVLSTR**SGE MVESIFGVSA**

B. MHLAQHAPGF LGLGNGLRPD AMHLRAPSTP PEAVTRSVSL ANIPGNEGTD SFRQDDIILT WALLQQRERG EDNPVEQFTW
GVRSVGGTET ILHFSLPALG LMLSRTTSGT VAAFLKAVQQ SIPSIEPSQL EAIFFYDEAP ISICPKGGES DTEPTEWTFQ
LRVLREGNIL NLWGLWPNKH VALSEQQAGD KLDTFIELLN IITQRPESPV R**ELLDPLPR**D LNQIWSWNAT LPPIIDRTMH
DIISEQAAAN LDKIALSSWD GQFTYAELET LSTSLGHHLC SLGITVGTIV PLCFEKSRWT IIALLGVMK**A GGAFALTDPT**
SQPEARLQAM VEQTGASLVV ASATQSELAR RLVPEDGEVV VVSEEFLASL SKKVDETASL PTIPTATSPL YIQFTSGSTG
KPKGVVISHA NYTSGAIPRA EAVGYNSSSR VFEFASYAFD VSIDCMLCTL AVGGTICIPS DADRMNDLGG AIGSSGANMA
HMTPSVARVL DPTVIAELDV LGLGGEAVSA ADAASWSKGK TSVIIAYGPS ECTVGCTVNN TFARHREERK LFTTGNIGKG
VGAVGWVADP EDHNRLVPVG SVGELLVEGP VVGIGYLGEP EKTAEVFIED PTWLVAGHGT VAGRHGRLYK TGDLVRYDPD
GSGDFVFVGR KDAQVKLRGQ RVELVEIEHH LRGKLPSGVK LAAEVINPIG GEPTLVTFVA EAPPHTESGR GPEGYPSFSD
EMVTALTGID EALGIELPRY MVPVAYLPLC EMPTLPSAKI DRKKLRAIGE TMTREQIAGI ARRSKRTESG APSTETERVL
QNVWRTLLGD QVDISVHDNF FALGGDSLRA MRLVPAARVM GIVLTVADVF RYPVLRDMAV VAKTVSIEQG RDAPDVVQPF
SLLENDWLPG QARAEASKHC GVDEADVEDV YPCTPLQEAL MALSAKVKDA YVAQRVLKLD SFDSADKLQA AFESIASDSA
ILRTRIIQVP QRGLVQVVVK EPILWRSAAT LTEYLEKDRE EQMDLGRPLV RYAMIREGRN VHFVLTMHHA VYDGWSMPLV
VDRVNQAYRG ILPRRPAAEF KHFIHYLNNR LDRVACDTYW RDQLAGATGV QFPRLPFEGY QTQADSLLEV DISLEGRRLP
TCPNATVTLA TVVRAAWALV ASQYCSGNKD IVFGETLTGR NAPIVGAEEI EGPMITTVPI RICIDRDIAV EEYLQSIAQQ
AVTQIPYEHA GLQHIRRLGD DALEACELRT GFVLHPAAGN VPADEQTPAN GLVPAGDSEA AQEALKFNTY ALMLVCSLSA
DGFFVMASFD SKTVDKGTMS RVLEQLRAVV HQLCEAEGKT VRVGDVQCLT DADREELQTL SWRMTLEGDN LAELGFVADD
IDGAWIVDAE SHEHPLPR**GA VGELLVR**TSK TLGAPAVAIE EPRWLKQAMA EGAQRGEGAR LYRTGRFASF DPVSIGNGGT
HTLRILKPSQ VKAQKAVVKR AAATAPAVSA TSTKQKTLRG IWSRLLKIDE AEIYLGDSFF TRGGDSITAM KLVSEARQQG
MQLTVAQVFA NRTLFEMANA MQPAPAPSAE RDVARTQVKA EYKPFSLFFN SLMERIRDTF ENKSWRIADI LPARPLQDIA
VQGTVELPRF SIRYELMHFE GMVDRARLFR ACQELVSRNE ILRTVFARLD GMCYSVVVEN PFVVPVVEYE IDGDDVESFA
AQLCRLDAQT RMPYGSSFVK WFFVTNGTRC SLVFRLSHAQ YDEICLPIFL NQLHQLYQDS DAVPVSHPFS AFVNHTLQDS
IPRAIPYWRD LLAGSPGISL LKPHTPVTNR RHFAIHRAFD ISARSRDVTV ATLPSAAWAL TLAR**LLGVND VVFGEVASGR**
SVDVPGIPDA NSITGPCWQY VPTRVRFGNL KTGHDLLAAV QHQHMMTSSH DCMGLTEIVR HCTDWDPKSV TWFDSVVHQD
VAHVETLSFL DRNARFETIY PYEEPLREWK IQAFHQGETL TLEIVTFESW KEHAVRLLDD LIVSMEQLVQ RPWEELNIVW
WA

Supplementary Fig. 8: Amino acid sequence. **A**. Amino acid sequence of L-ornithine N(5)-monooxygenase SidA. **B**. Amino acid sequence of Non-ribosomal peptide synthetase SidD.

Supplementary Fig. 9

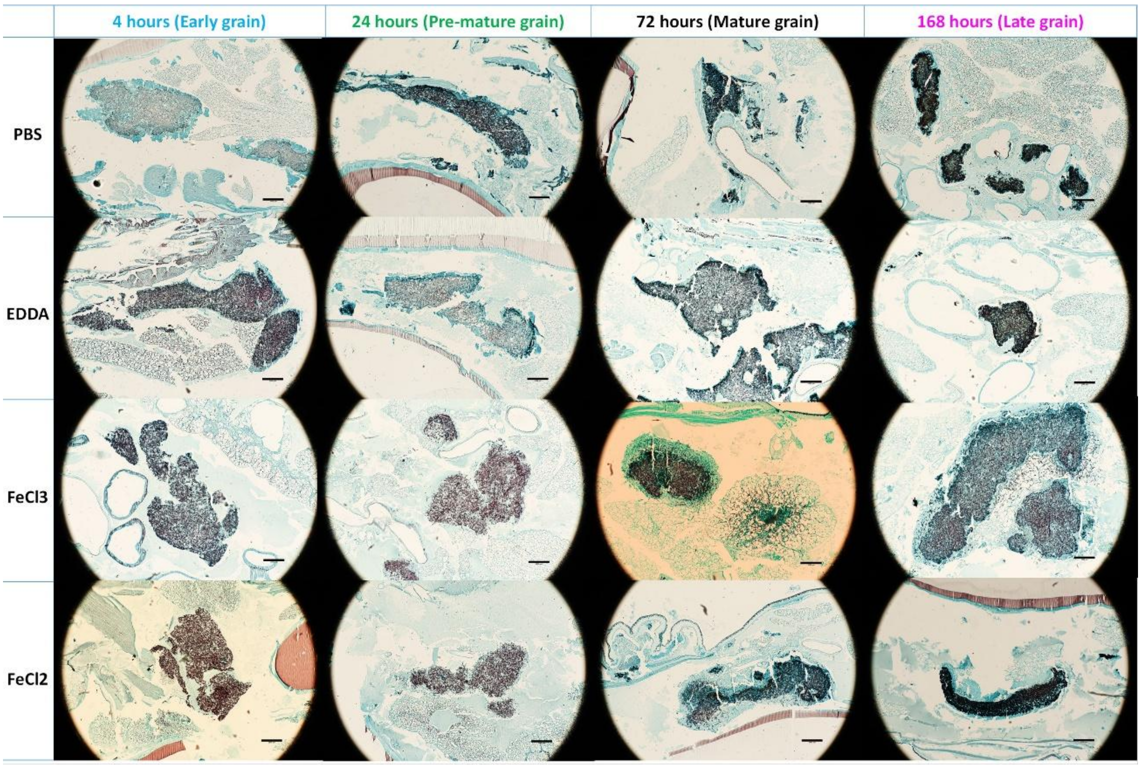

Supplementary Fig. 9: The influence of changing iron conditions on *M. mycetomatis* Grain development over time in *G. mellonella* larvae visualized 100 times magnified using Grocott staining.

Supplementary Fig. 10

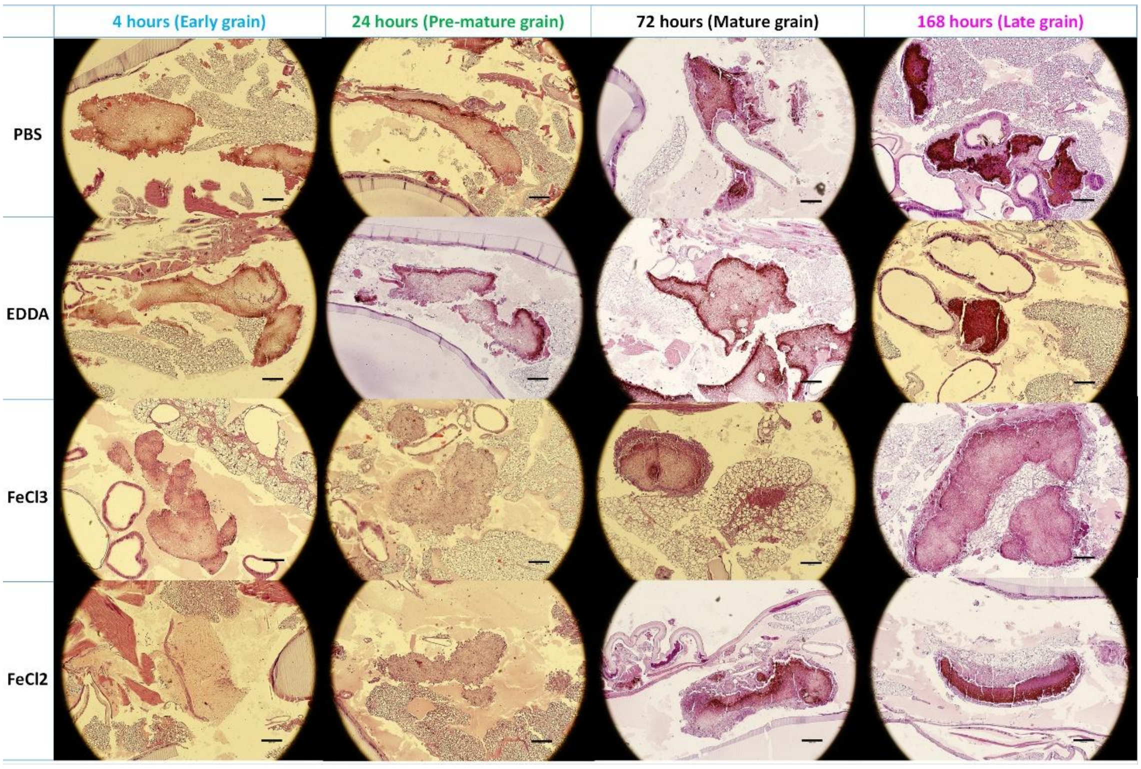

Supplementary Fig. 10: The influence of changing iron conditions on *M. mycetomatis* Grain development over time in *G. mellonella* larvae visualized 100 times magnified using H&E staining.

Supplementary Fig. 11

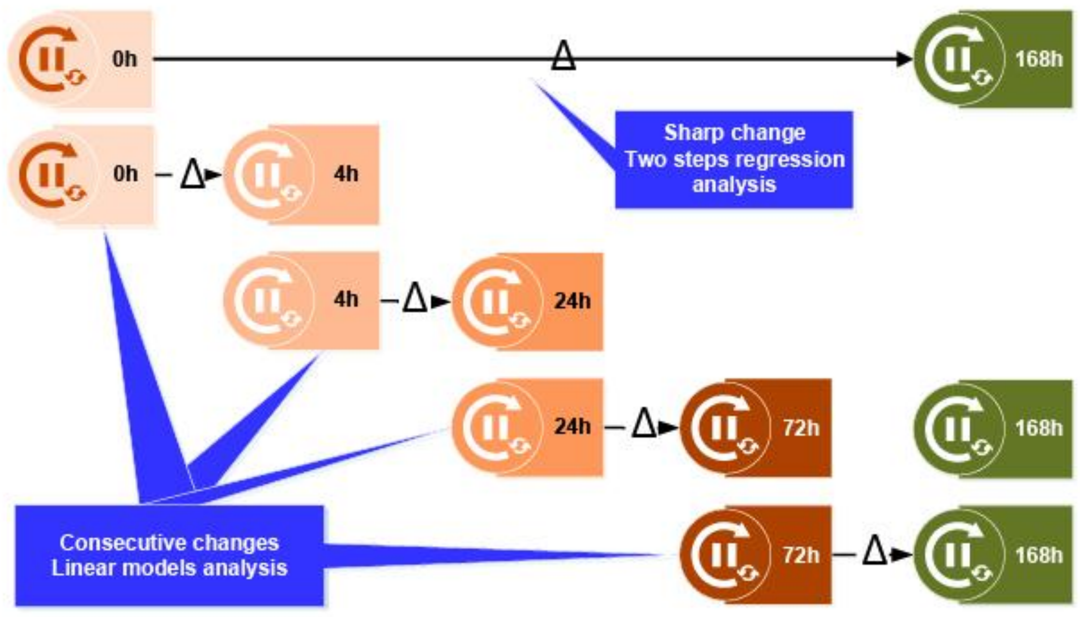

Supplementary Fig. 11: DEA methodology. Two types of changes are investigated. Sharp change between time point 0h and 168h, and consecutive changes between time points 0h & 4h, 4h &24h, 24h & 72h, and 72h & 168h.

Supplementary Fig. 12

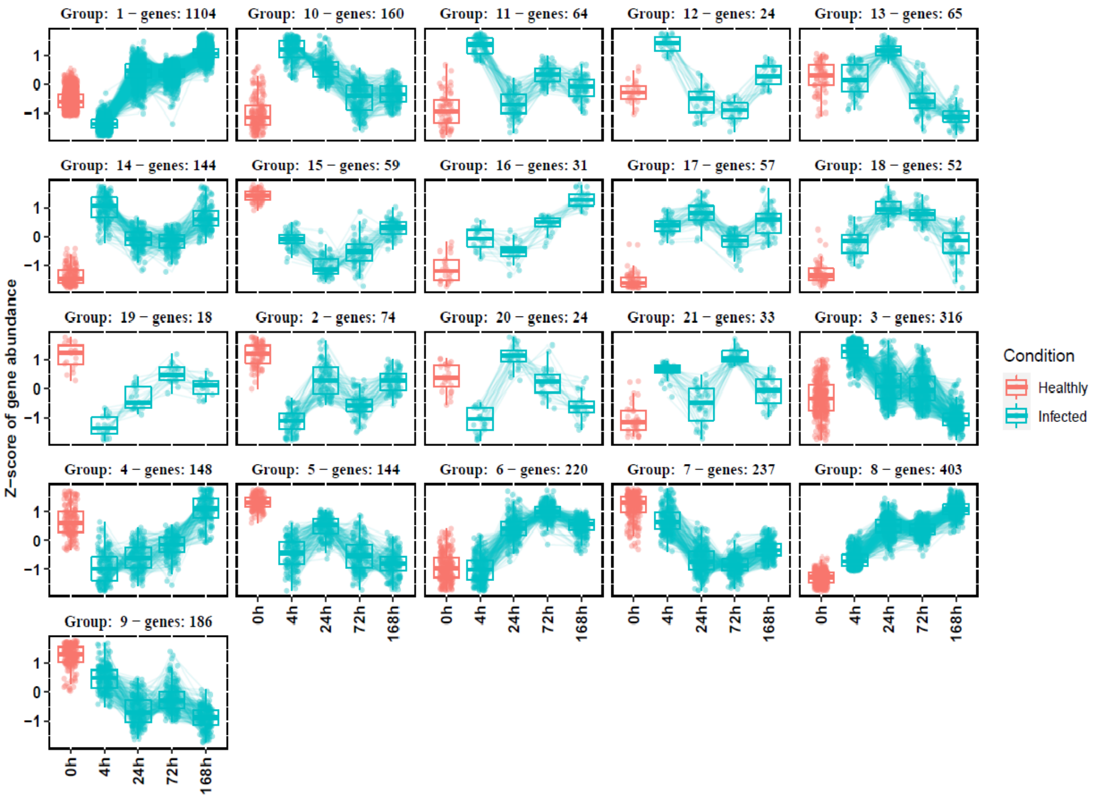

Supplementary Fig. 12: DEG patterns for *G. mellonella* larvae RNA-Seq reads mapped to host genome. Source data are available in the Gene Expression Omnibus (GEO) under accession number GSE213329.

Supplementary Fig. 13

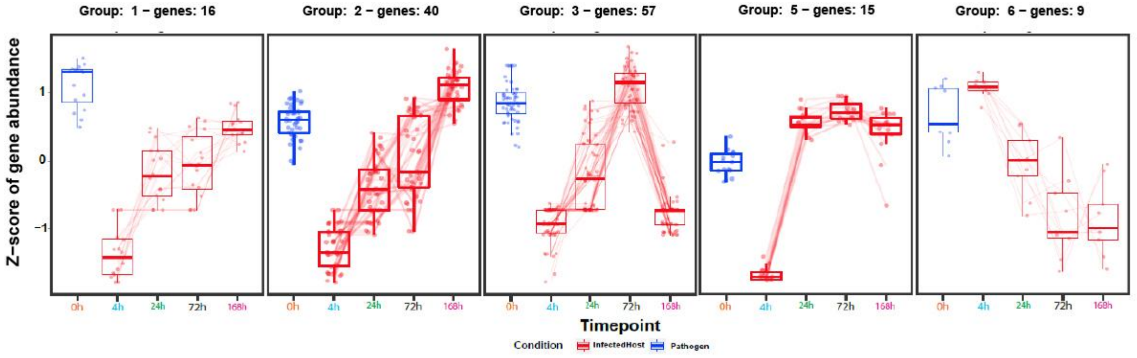

Supplementary Fig. 13: DEG patterns for *G. mellonella* larvae RNA-Seq reads mapped to pathogen genome. Source data are available in the Gene Expression Omnibus (GEO) under accession number GSE213329.

Supplementary Fig. 14

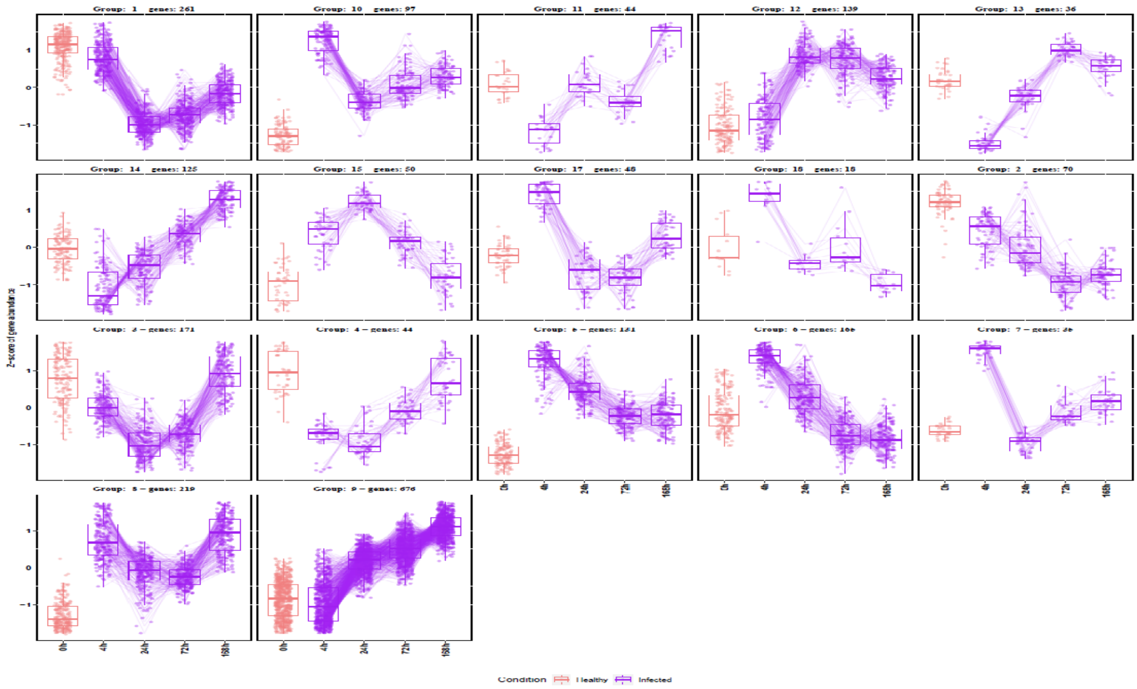

Supplementary Fig. 14: DEG TSS patterns were analyzed for *G. mellonella* larvae reads mapped to host genome. Source data are available in the Gene Expression Omnibus (GEO) under accession number GSE213332.

Supplementary Note [1] : Total RNA QC and total tag count (Run I)

1.1 NanoDrop QC

The quality of the total RNA was measured using NanoDrop, all samples passed the QC threshold required for RNA-seq. The Nanodrop QC report shown below.

Library Name	conc (ug/ul)	260/280	260/230	volume (ul)	amount (ug)	volume (ul)
**T0_Galleria_RNA_1**	0.29	2.14	2.24	39.50	11.65	42.00
**T0_Galleria_RNA_2**	0.05	2.06	0.94	31.50	1.70	35.00
**T0_Galleria_RNA_3**	0.15	2.14	1.37	37.50	5.65	40.00
**T0_MM55_RNA_1**	0.74	2.18	2.13	37.50	27.56	40.00
**T0_MM55_RNA_2**	0.65	2.18	2.36	33.50	21.87	36.00
**T0_MM55_RNA_3**	0.95	2.18	1.82	39.50	37.48	42.00
**T4_infected_Galleria_RNA_1**	0.13	2.12	1.05	32.50	4.08	35.00
**T4_infected_Galleria_RNA_2**	0.27	2.12	2.02	32.50	8.87	35.00
**T4_infected_Galleria_RNA_3**	0.08	2.10	0.42	31.50	2.46	35.00
**T24_infected_Galleria_RNA_1**	0.16	2.14	0.65	26.50	4.36	29.00

**T24_infected_Galleria_RNA_2**	0.26	2.14	0.76	26.50	6.85	29.00
**T24_infected_Galleria_RNA_3**	0.29	2.14	2.23	26.50	7.57	29.00
**T72_infected_Galleria_RNA_1**	0.17	2.13	2.02	26.50	4.62	29.00
**T72_infected_Galleria_RNA_2**	0.33	2.14	1.76	26.50	8.69	29.00
**T72_infected_Galleria_RNA_3**	0.13	2.13	0.51	26.50	3.48	29.00
**T7d_infected_Galleria_RNA_1**	0.18	2.14	1.65	26.50	4.79	29.00
**T7d_infected_Galleria_RNA_2**	0.21	2.13	2.11	26.50	5.45	29.00
**T7d_infected_Galleria_RNA_3**	0.16	2.12	0.67	26.50	4.13	29.00

1.2 Bioanalyzer RIN value

In addition to NanoDrop RNA QC confirmed with Bioanalyzer, RIN values are shown below

Library Name	RIN
**T0_Galleria_RNA_1**	9.3
**T0_Galleria_RNA_2**	8.8
**T0_Galleria_RNA_3**	9.0
**T0_MM55_RNA_1**	7.4
**T0_MM55_RNA_2**	7.2
**T0_MM55_RNA_3**	7.1
**T4_infected_Galleria_RNA_1**	8.5
**T4_infected_Galleria_RNA_2**	9.1
**T4_infected_Galleria_RNA_3**	8.1
**T24_infected_Galleria_RNA_1**	8.1
**T24_infected_Galleria_RNA_2**	9.5
**T24_infected_Galleria_RNA_3**	9.5
**T72_infected_Galleria_RNA_1**	9.3
**T72_infected_Galleria_RNA_2**	8.7
**T72_infected_Galleria_RNA_3**	9.3
**T7d_infected_Galleria_RNA_1**	9.6
**T7d_infected_Galleria_RNA_2**	9.3
**T7d_infected_Galleria_RNA_3**	9.4

1.3 Total tag count of the RNA-seq reads

Total tag count computed from all Fastq files from RNA-seq

Library Name	Tag count

**T0_Galleria_RNA_1.fastq.gz**	51,320,499
**T0_Galleria_RNA_2.fastq.gz**	50,139,792
**T0_Galleria_RNA_3.fastq.gz**	47,776,417
**T0_MM55_RNA_1_.gz**	51,110,468
**T0_MM55_RNA_2.fastq.gz**	50,547,149
**T0_MM55_RNA_3.fastq.gz**	50,990,095
**T24_infected_Galleria_RNA_1.fastq.gz**	46,911,292
**T24_infected_Galleria_RNA_2.fastq.gz**	45,810,500
**T24_infected_Galleria_RNA_3.fastq.gz**	50,446,724
**T4_infected_Galleria_RNA_1.fastq.gz**	50,752,091
**T4_infected_Galleria_RNA_2.fastq.gz**	52,431,178
**T4_infected_Galleria_RNA_3.fastq.gz**	52,649,737
**T72_infected_Galleria_RNA_1.fastq.gz**	47,582,236
**T72_infected_Galleria_RNA_2.fastq.gz**	50,126,726
**T72_infected_Galleria_RNA_3.fastq.gz**	47,034,937
**T7d_infected_Galleria_RNA_1.fastq.gz**	47,951,226
**T7d_infected_Galleria_RNA_2.fastq.gz**	48,070,777
**T7d_infected_Galleria_RNA_3.fastq.gz**	48,878,244

Supplementary Note [2] : LQ-ssCAGE

2.1 LQ-ssCAGE library preparation

LQ-ssCAGE sequencing libraries were prepared following the procedures described by Takahashi and colleagues[2]. Similar to RNA-Seq, LQ-ssCAGE was prepared for both the *G. mellonella* larvae samples and *M. mycetomatis* with 25 ng as minimum total RNA input.

2.2 LQ-ssCAGE library sequencing

The same HiSeq2500 instrument was used as described before but with Paired-End; 50base. After performing base calling for all samples, we returned 217,641,602 total tags for the *G. mellonella* larvae samples and 68,428,840 total tags for the *M. mycetomatis* samples. Total tags count per LQ-ssCAGE library are shown below.

Library Name	Tag count
**T0_Galleria_RNA_1.fastq.gz**	18,936,800
**T0_Galleria_RNA_2.fastq.gz**	7,111,918
**T0_Galleria_RNA_3.fastq.gz**	14,301,764
**T0_MM55_RNA_1_.gz**	24,609,382

**T0_MM55_RNA_2.fastq.gz**	21,267,229
**T0_MM55_RNA_3.fastq.gz**	22,552,229
**T24_infected_Galleria_RNA_1.fastq.gz**	15,232,719
**T24_infected_Galleria_RNA_2.fastq.gz**	15,514,884
**T24_infected_Galleria_RNA_3.fastq.gz**	13,889,253
**T4_infected_Galleria_RNA_1.fastq.gz**	17,173,635
**T4_infected_Galleria_RNA_2.fastq.gz**	15,515,125
**T4_infected_Galleria_RNA_3.fastq.gz**	6,646,437
**T72_infected_Galleria_RNA_1.fastq.gz**	17,419,374
**T72_infected_Galleria_RNA_2.fastq.gz**	15,817,270
**T72_infected_Galleria_RNA_3.fastq.gz**	14,767,851
**T7d_infected_Galleria_RNA_1.fastq.gz**	15,235,936
**T7d_infected_Galleria_RNA_2.fastq.gz**	15,311,786
**T7d_infected_Galleria_RNA_3.fastq.gz**	14,766,850

2.3 LQ-ssCAGE library mapping

The same mapping strategy was used for LQ-ssCAGE as for RNA-seq described above. For each of the *M. mycetomatis* libraries, on average, 22.8 million reads were mapped, of these, on average, 35.0% uniquely mapped to the ASM127576v2 genome. For each of the *G. mellonella* larvae libraries, on average, 14.5 million reads were mapped, of these, on average, 47.0% were uniquely mapped against ASM364042v2. We tested the correlation between the RNA-Seq and LQ-ssCAGE (Supplementary Fig. 15)

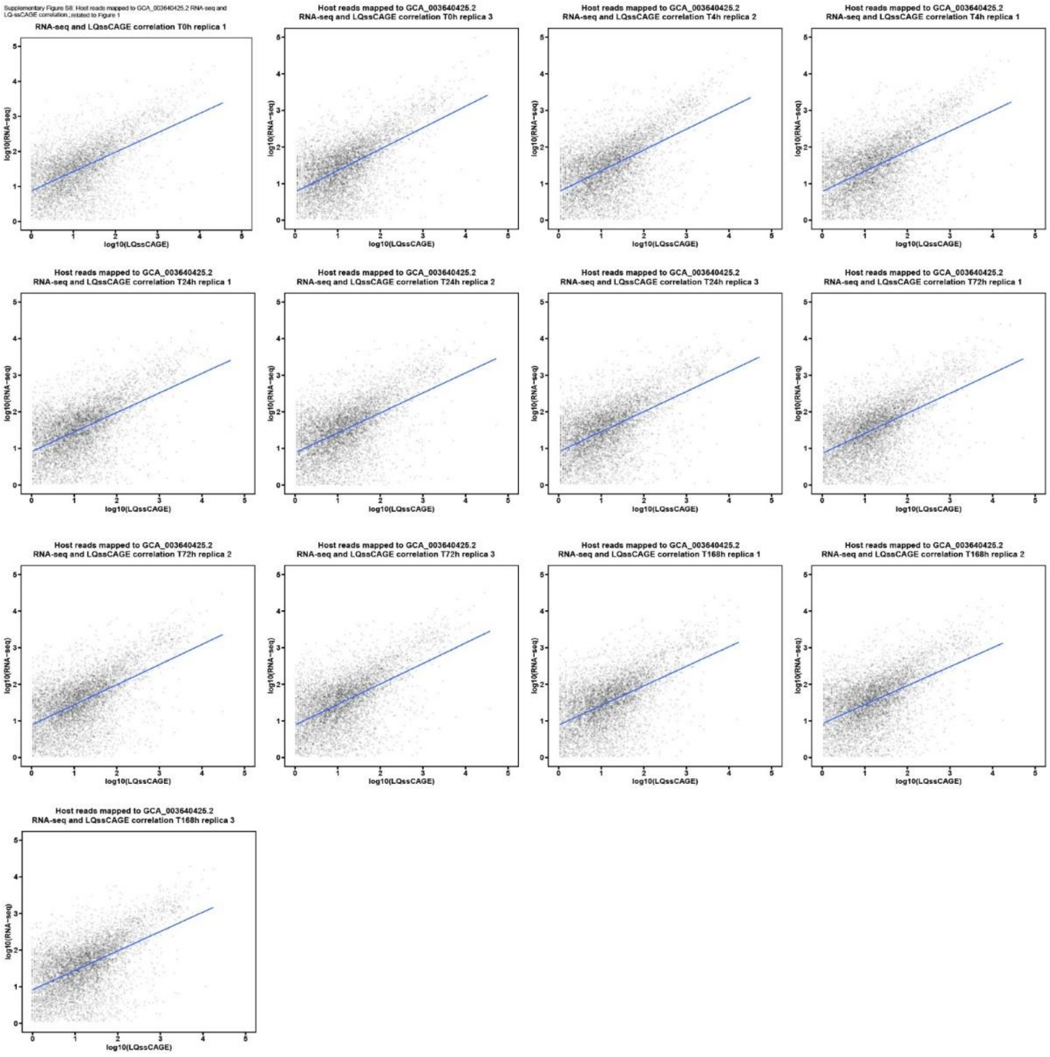

Supplementary Fig. 15: Correlation between the RNA-Seq and LQ-ssCAGE. Source data are available in the Gene Expression Omnibus (GEO) under accession number GSE213329 and GSE213332.

2.4 LQ-ssCAGE TSS peaks calling

Using LQ-ssCAGE, a genome-wide map of Transcription start site (TSS) was established for the *Galleria mellonella* larvae and *M. mycetomatis* by precisely identifying promoters and enhancers. To identify the promoters and enhancers for the two species, we created two Biostrings-based genome data packages. The two Bioconductor packages used by CAGEfightR to identify TSS and enhancers[3]. The two packages were used for annotation of the CAGE-defined TSSs and gene-level expression. The mapped reads for each library were converted to CAGE tags (CTSSs) in BED format using an in-house script. The CTSS tag clusters (TC) were identified from the CTSSs and used for the subsequent analysis and quantification of the LQ-ssCAGE data. In short, for CTSS, counts were normalized using Tags-per-Million (TPM), and the normalized CTSS counts were used to calculate the pooled CTSS (CTSS signal across all samples). The pooled CTSS were further processed to reduce single tags spread across the genome. This is accomplished by removing CTSSs detected in only a single or few samples. The final set of the pooled CTSSs was used for the analysis at the level of clusters of CTSSs (Promoters and enhancers identification) and analysis at the level of annotated genes.

2.5 LQ-ssCAGE Prediction of the transcription factors binding sites (TFBS) and motif activity overtime

To predict transcription factors binding sites (TFBS) for the *G. Mellonella*, we used findMotifsGenome.pl from Homer P[4]. The input for the command is the set of identified Promoters and Enhancers from LQ-ssCAGE data. Also, the Homer command requires a FAST and GFF file for the *G. mellonella* genome, which was obtained from NCBI.

Supplementary Note [3] : G. mellonella promoters and active enhancers expression landscape during infection

3.1 TSS and enhancers

LQ-ssCAGE enabled mapping of the transcription start sites (TSS) and, therefore, the regulatory elements (Promoters and active enhancers)[5]. The mapped TSS of the *G. mellonella* larvae libraries were used for identifying promoters and active enhancers. Based on the CAGE tags generated by LQ-ssCAGE, we defined (16,548) expressed TSS from all libraries (n=16). We found that 43.1% (7,129) of the defined *G. mellonella* TSS are promoters followed by 15.1% (2,495) Coding Sequence (CDS), 13.3% (2,209) intergenic and 10.5% (1,731) intron (Supplementary Fig. 16). Additionally, LQ-ssCAGE enabled the identification of 11,031 active enhancers in *G. mellonella*. Of these defined bidirectional transcribed enhancers, 34.5% (6,466) were overlapping intronic regions, and 24.4% (4,565) were overlapping intergenic regions (Supplementary Fig. 16). The principal component analysis of the defined *G. mellonella* LQ-ssCAGE TSS (Supplementary Fig. 2) separated the healthy larvae from the infected larvae samples and the samples per time point (grain development stage). We found that all defined TSS were expressed in healthy and infected larvae (Supplementary Fig. 16).

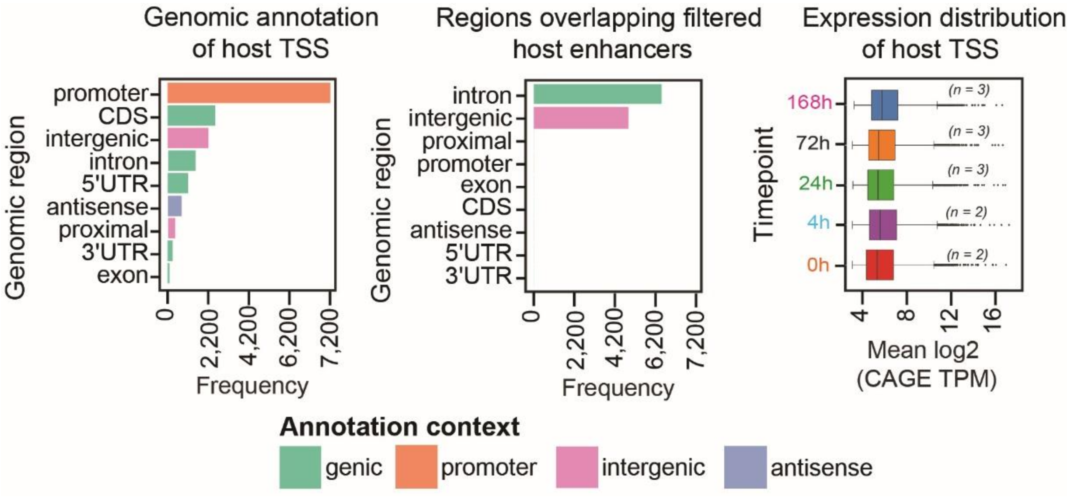

Supplementary Fig. 16: Landscape of the promoters and active enhancers of the host genome. Source data are available in the Gene Expression Omnibus (GEO) under accession number GSE213332.
Barplots of genomic annotation of the TSS and the regions overlapping filtered enhancers. The bars are colored by annotation context. The majority of the identified TSS peaks are annotated as promoters. Active enhancers are annotated as intronic and intergenic regions. The expression distribution of TSS shows minor differences of expression per time point illustrated. Expression distribution of host TSS represented as boxplot with lower whisker (represents smallest observation greater than or equal to lower hinge in the boxplot - 1.5 * IQR), median and upper whisker (represents largest observation less than or equal to upper hinge in the boxplot + 1.5 * IQR).The largest mean CAGE TPM expression was observed at 168h.

3.2 differential expression of the host TSS

To understand the role of differential expression of the TSS and active enhancers, we used edegR (Methods). We observed that of the total 16,548 *G. mellonella* TSS, only 3,279 TSS were significantly differentially expressed (Supplementary Data 9**)**. The most differentially expressed TSS are down-regulated between T4h-T168h. A summary of the up and down-regulated TSS and active enhancers is shown in (Supplementary Fig. 17).

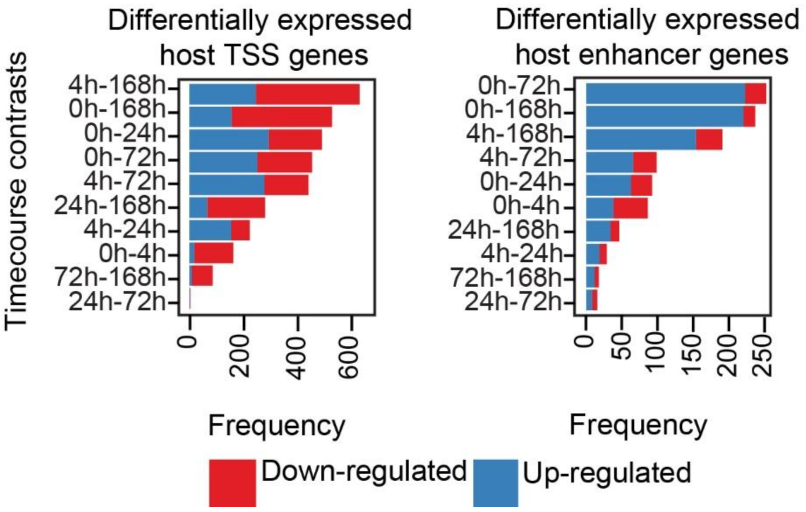

Supplementary Fig. 17: Differential expressions of the host TSS and enhancers. Source data are available in the Gene Expression Omnibus (GEO) under accession number GSE213332.

3.3 TFBS prediction and motif analysis

To identify potential transcription factor binding sites (TFBSs) in the *G. mellonella* larvae genomes, we used HOMER Motif tools[4]. Using the promoter regions, we predicted 28 transcription factors. We performed motif activity analysis to analyze trans-regulatory elements that regulate distant genes.

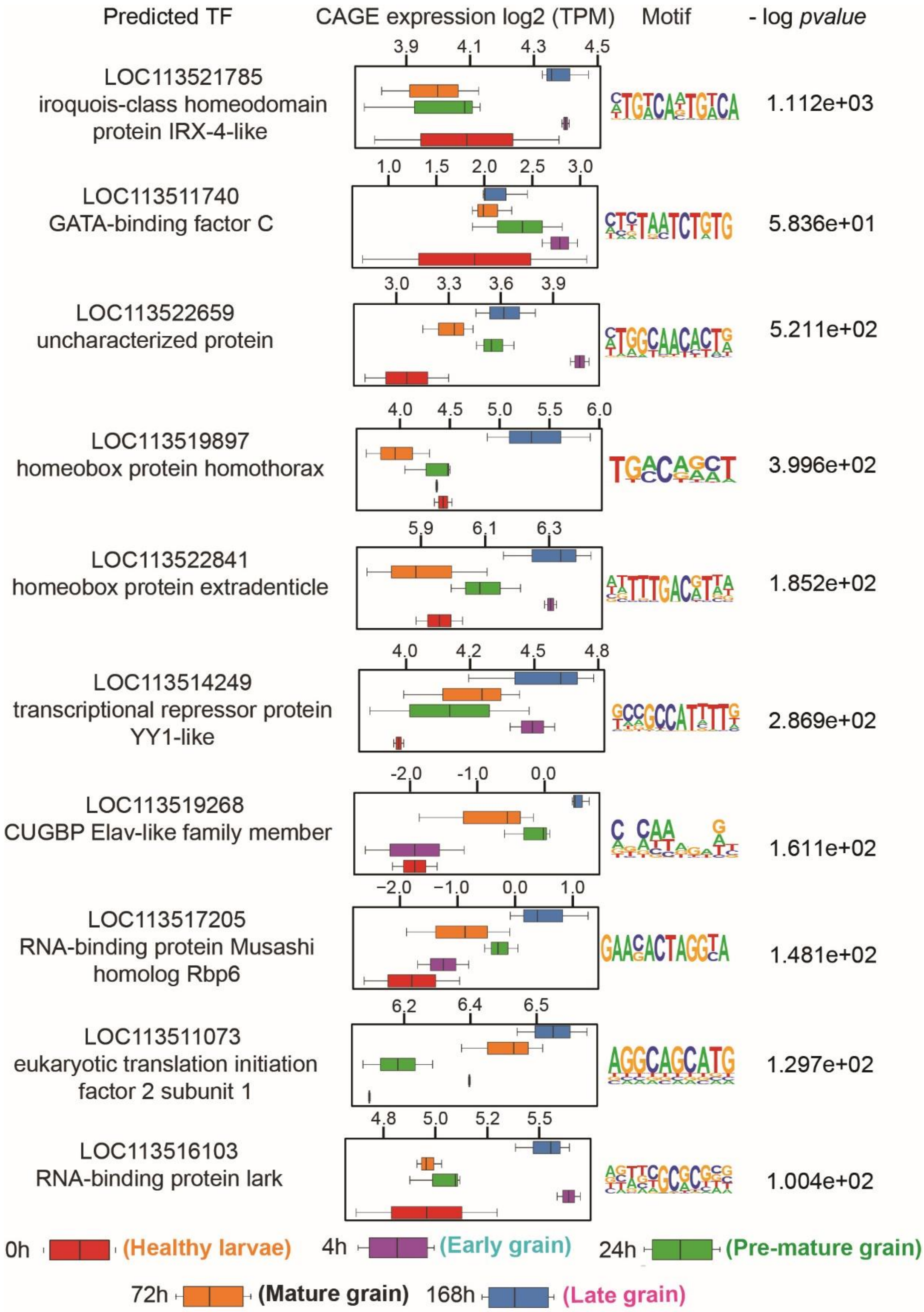

Supplementary Fig. 18: Predicted TF and motif activity analysis. Bar represents the SEM. Source data are available in the Gene Expression Omnibus (GEO) under accession number GSE213332.

Predicted transcription factors (TF). TF predicted using Homer motif analysis (Material and Methods) for each of the TF CAGE expressions and colored by time point. The CAGE expression

shown as boxplot with lower whisker (represents smallest observation greater than or equal to lower hinge in the boxplot - 1.5 * IQR), median and upper whisker (represents largest observation less than or equal to upper hinge in the boxplot + 1.5 * IQR). Sequence log for each motif is shown together with the -log *p-value*. Full list of all predicted TF and associated GO terms in (Supplementary Data 10).

The motif activities (Supplementary Fig. 18) represent the average expression level of genes with a predicted binding site for each motif. Figure 3B shows the top 10, broadly expressed transcription factors (TF). The TFs in Supplementary Fig. 18.

are ordered by the -log p-value, as an example, the TF LOC113521785 (iroquois-class homeodomain protein IRX-4-like) and (LOC113511740) GATA-binding factor C are highly expressed in at T4h (pre-mature grain stage). The gene ontology (GO) biological process associated with these genes is the regulation of transcription. The motif activity analysis indicates that at the time of the infection of the larvae, TFs altered their activity based on the eumycetoma grain development stage. A list of all predicted TF and associated GO terms is provided in Supplementary Data 3.

Supplementary Note [4] : Total RNA QC and total tag count (Run II)

4.1 RNA quantification and library DNA

	RNA Quantitation･QC		Library DNA		
**Sample Name**	【Ribogreen】 RNA concentration (ng/uL)	【RNA QC】 RIN score	Input Amount RNA (ng)	Library DNA Average Size (bp)	Library DNA concentration(qPCR) (nM)
**OP00043-001**	52.80	8.2	20	349	10.63
**OP00043-002**	24.47	8.7	20	370	9.15
**OP00043-003**	42.40	8.5	20	378	10.34
**OP00043-004**	28.77	8.1	20	365	9.09
**OP00043-005**	64.01	7.6	20	366	6.35
**OP00043-006**	16.32	7.5	20	390	6.59
**OP00043-007**	50.31	8	20	374	6.57
**OP00043-008**	14.85	7.8	20	359	9.28
**OP00043-009**	6.21	8.6	20	375	5.76
**OP00043-010**	26.00	9.5	20	397	12.72

**OP00043-011**	1.65	9.8	20	376	22.71
**OP00043-012**	12.25	9.2	20	375	40.53
**OP00043-013**	6.17	9.3	20	361	11.86
**OP00043-014**	3.44	9.5	20	379	23.47
**OP00043-015**	7.79	9.3	20	379	18.75
**OP00043-016**	127.44	9.1	20	369	11.16
**OP00043-017**	64.74	9	20	356	19.59
**OP00043-018**	0.81	7.9	20	382	20.29
**OP00043-019**	167.37	8.3	20	371	2.59
**OP00043-020**	34.89	8	20	391	8.99
**OP00043-021**	47.78	8.2	20	392	9.01
**OP00043-022**	119.92	8.5	20	348	3.22
**OP00043-023**	26.93	7.5	20	352	10.67
**OP00043-024**	37.41	8	20	346	14.99
**OP00043-025**	50.47	7.7	20	355	12.97
**OP00043-026**	46.29	7.3	20	372	13.10
**OP00043-027**	159.34	8.7	20	352	15.11
**OP00043-028**	34.00	7.7	20	376	13.08
**OP00043-029**	36.32	7.8	20	365	18.98
**OP00043-030**	137.83	8.7	20	350	34.48
**OP00043-031**	71.36	8.2	20	347	76.13
**OP00043-032**	53.08	7.9	20	347	56.62
**OP00043-033**	55.26	8.2	20	365	19.59
**OP00043-034**	76.72	6.9	20	368	12.15
**OP00043-035**	73.19	7.7	20	372	13.46
**OP00043-036**	37.80	8.0	20	358	14.11

4.2 Total tag count and mapping rate

Sample Name	Total tag count	Reads mapped to host only	Reads mapped to pathogen only
**OP00043-001**	30,973,214	27,028,959	3,548
**OP00043-002**	34,302,779	30,228,292	3,109
**OP00043-003**	31,334,533	27,714,688	2,332
**OP00043-004**	32,497,656	28,652,495	3,118
**OP00043-005**	29,474,419	26,081,358	2,466
**OP00043-006**	37,607,680	33,690,637	2,349
**OP00043-007**	35,301,978	31,501,077	2,660
**OP00043-008**	41,557,771	37,455,955	2,796
**OP00043-009**	39,510,938	35,821,123	1,469
**OP00043-010**	30,669,657	27,300,255	15,441
**OP00043-011**	43,144,917	38,389,958	21,402
**OP00043-012**	44,413,951	39,211,952	20,199
**OP00043-013**	25,223,358	22,360,365	8,484
**OP00043-014**	30,569,339	26,895,227	9,276
**OP00043-015**	42,041,764	37,065,550	13,597
**OP00043-016**	40,779,249	35,767,278	11,908

**OP00043-017**	30,672,625	26,968,520	9,833
**OP00043-018**	32,102,972	28,639,457	9,643
**OP00043-019**	41,373,630	34,194,433	23,993
**OP00043-020**	40,286,581	35,536,005	17,167
**OP00043-021**	44,848,158	39,637,525	21,384
**OP00043-022**	32,362,667	23,953,349	12,381
**OP00043-023**	34,153,371	30,081,722	12,483
**OP00043-024**	35,733,991	31,102,413	15,037
**OP00043-025**	32,983,529	28,384,540	23,171
**OP00043-026**	32,864,201	28,210,398	14,129
**OP00043-027**	36,167,617	31,131,387	32,035
**OP00043-028**	37,068,665	32,525,701	17,775
**OP00043-029**	33,966,894	29,511,839	26,391
**OP00043-030**	30,661,861	26,474,516	29,125
**OP00043-031**	38,742,209	33,880,834	57,608
**OP00043-032**	44,762,595	38,776,736	69,955
**OP00043-033**	41,445,053	36,344,936	43,014
**OP00043-034**	29,449,799	25,803,958	17,926
**OP00043-035**	30,377,944	26,194,773	17,353
**OP00043-036**	43,634,077	37,788,672	20,638

4.3 UMAP clustering of the samples

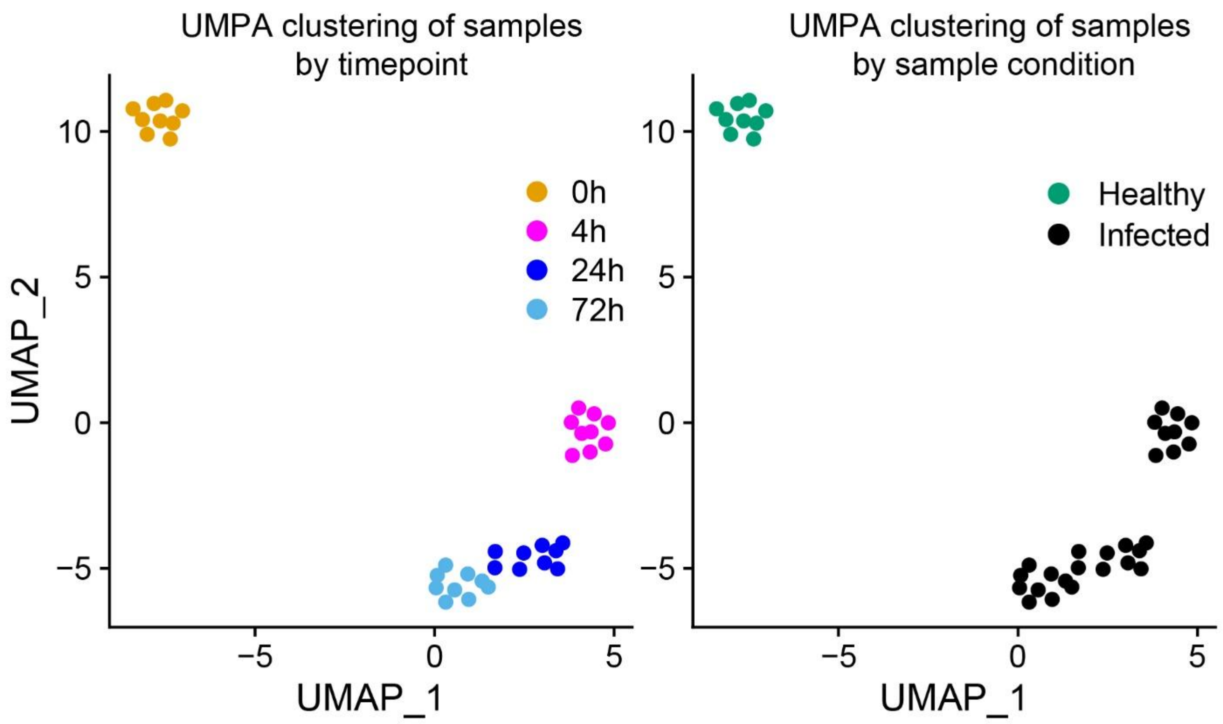

Supplementary Fig. 19: UMAP clustering of the sample (n=36). The UMAP shows perfect clustering of the sample per timepoint and experimental conditions. Source data are available in the Gene Expression Omnibus (GEO) under accession number GSE280443.

Supplementary Note [5] : Obtaining *A. fumigatus* homologous genes in *M. mycetomatis*

5.1 BLAST

Firstly, the protein sequence of *A. fumigatus* genes was retrieved from the UniProt database[6]. To obtain the homologous genes in *M. mycetomatis,* we used the NCBI protein-protein BLAST (blastp) web service[7]. Blastp run using the following parameters for the chosen search set: The database was non-redundant

protein sequences(nr), and the organism used *M. mycetomatis* (NCBI: taxid:100816).

5.2 Protein–Protein-Interaction network

String stores and provides known and predicted protein-protein interactions (PPI) for thousands of organisms. The protein-protein interactions for homologous genes in *M. mycetomatis* enabled us to infer other genes that are interacting with the homologous genes in the PPI. In the PPI, we used the minimum required interaction score = low confidence (0.150) and kept the default value for the rest of the parameters. For each PPI, we obtained the PPI enrichment p-value, the functional enrichments in the PPI (Gene Ontology terms and KEGG pathways), and network characteristics (hub genes, number of nodes, number of edges). Finally, we analysed and visualized the gene expression of homologous genes in *M. mycetomatis* in the list of the differentially expressed genes from RNA-Seq.

Supplementary Note [6] : Proteomic analysis of *M. mycetomatis* mycelia

6.1protein preparation for LC-MS/MS analysis

20 µg of protein sample and 200 µL of 50 mM ammonium bicarbonate, 8 M Urea pH 7.8 were transferred to a 30 kDa MWCO HY spin filter (Sartorius) and vortexed and centrifuged at 14,000 g for 20 minutes. 200 µL of 50 mM ammonium bicarbonate, 8 M Urea pH 7.8 was transferred to each sample filter which were then vortexed and centrifuged at 14,000 g for 20 minutes. 100 µL of 5 mM TCEP was transferred to each sample filter, vortexed and incubated at room temperature for 20 minutes. 3 µL of 500 mM iodoacetamide was transferred to each sample filter, vortexed and incubated in the dark for 20 minutes and then centrifuged at 14,000 g for 20 minutes. Sample filters were then washed twice by transferring 100 µL of 50 mM ammonium bicarbonate, 8 M urea pH 7.8 to each sample filter, vortexing and then centrifuging at 14,000 g for 20 minutes. Two sequential washes were carried out by transferring 100 µL of 50 mM ammonium bicarbonate pH 7.8 to each sample filter, vortexing and centrifuging at 14,000 g for 20 minutes. 60 µL of 50 mM ammonium bicarbonate pH 7.8, 2.5 µL trypsin (0.4 µg/µL) and 1 µL of (1% (w/v)) ProteaseMax were transferred to the sample filters, vortexed and incubated for 18 hours at 37°C. After incubation, filters were transferred to new collection tubes and centrifuged at 14,000 g for 20 minutes. 50 µL of 50 mM ammonium bicarbonate pH 7.8 was transferred to each sample filter, vortexed and centrifuged at 14,000 g for 20 min. 80 µL of the eluent was transferred to a fresh 1.5 mL tube and 20 µL of 5 × LC-MS/MS loading buffer (20% (v/v) acetonitrile, 2% (v/v) trifluoroacetic acid in HPLC grade water) was added, and the solution was mixed by vortexing. Tubes were labelled with sample ID and stored at -20°C for up to six weeks.

Supplementary References

1 Heinekamp, T. *et al.* Aspergillus fumigatus melanins: interference with the host endocytosis pathway and impact on virulence. *Front Microbiol* **3**, 440 (2012). https://doi.org/10.3389/fmicb.2012.00440

2 Takahashi, H., Nishiyori-Sueki, H., Ramilowski, J. A., Itoh, M. & Carninci, P. Low Quantity Single Strand CAGE (LQ-ssCAGE) Maps Regulatory Enhancers and Promoters. *Methods Mol Biol* **2351**, 67-90 (2021).

3 Thodberg, M., Thieffry, A., Vitting-Seerup, K., Andersson, R. & Sandelin, A. CAGEfightR: analysis of 5'-end data using R/Bioconductor. *BMC Bioinformatics* **20**, 487 (2019).

4 Heinz, S. *et al.* Simple combinations of lineage-determining transcription factors prime cis-regulatory elements required for macrophage and B cell identities. *Mol Cell* **38**, 576-589 (2010).

5 Arner, E. *et al.* Transcribed enhancers lead waves of coordinated transcription in transitioning mammalian cells. *Science* **347**, 1010-1014 (2015). https://doi.org/10.1126/science.1259418

6 UniProt, C. UniProt: the universal protein knowledgebase in 2021. *Nucleic Acids Res* **49**, D480-D489 (2021).

7 Altschul, S. F., Gish, W., Miller, W., Myers, E. W. & Lipman, D. J. Basic local alignment search tool. *J Mol Biol* **215**, 403-410 (1990).
