# Supplementary material for: Iron regulatory pathways differentially expressed during Madurella mycetomatis grain development in Galleria mellonella
Source: Nat Commun. 2025 Jun 25;16:5324. doi: 10.1038/s41467-025-60875-2 (PMC12198395; doi:10.1038/s41467-025-60875-2)
Supplement: Supplementary file 2 — Description of Additional Supplementary Files [file 41467_2025_60875_MOESM2_ESM.pdf]

Description of Additional Supplementary Files:

Supplementary Data 1: Analysis of the RNA-seq reads mapped to the host and the pathogen.

Supplementary Data 2: List of selected gene with their primer pairs and sequence.

Supplementary Data 3: Annotation of the host top 50 DEG, related to Figure 3.

Supplementary Data 4: Annotation of the pathogen top 20 DEG, related to Figure 4.

Supplementary Data 5: Host DEG patterns annotated with UniProt annotation, related to Figure 5 A.

Supplementary Data 6: Pathogen DEG patterns annotated with UniProt annotation, related to Figure 5 C.

Supplementary Data 7: Homologues of A. fumigatus DHN-melanin and pyomelanin biosynthesis pathways in M. mycetomatis.

Supplementary Data 8: List of homologues involved in the iron regulation of D. melanogaster in G. mellonella with their statistical report from NCBI blast.

Supplementary Data 9: List of differentially expressed TSS. For testing differentially expressed genes, F-statistic, the associated P-value, and the adj. P-value were corrected using Benjamini-Hochberg multiple testing correction.

Supplementary Data 10: List of all predicted TF and associated GO terms. The associated log *P-value*, were computer by HOMER Motif Discovery and Analysis.
