# Supplementary material for: Iron regulatory pathways differentially expressed during Madurella mycetomatis grain development in Galleria mellonella
Source: Nat Commun. 2025 Jun 25;16:5324. doi: 10.1038/s41467-025-60875-2 (PMC12198395; doi:10.1038/s41467-025-60875-2)
Supplement: Supplementary file 13 — Reporting Summary [file 41467_2025_60875_MOESM13_ESM.pdf]

nature portfolio

Corresponding author(s): Imad Abugessaisa & Wendy W.J. van de Sande

Last updated by author(s): May 15, 2025

Reporting Summary

Nature Portfolio wishes to improve the reproducibility of the work that we publish. This form provides structure for consistency and transparency in reporting. For further information on Nature Portfolio policies, see our Editorial Policies and the Editorial Policy Checklist.

Statistics

For all statistical analyses, confirm that the following items are present in the figure legend, table legend, main text, or Methods section.

n/a	Confirmed	
☐	☒	The exact sample size (*n*) for each experimental group/condition, given as a discrete number and unit of measurement
☐	☒	A statement on whether measurements were taken from distinct samples or whether the same sample was measured repeatedly
☐	☒	The statistical test(s) used AND whether they are one- or two-sided<br>*Only common tests should be described solely by name; describe more complex techniques in the Methods section.*
☒	☐	A description of all covariates tested
☐	☒	A description of any assumptions or corrections, such as tests of normality and adjustment for multiple comparisons
☐	☒	A full description of the statistical parameters including central tendency (e.g. means) or other basic estimates (e.g. regression coefficient) AND variation (e.g. standard deviation) or associated estimates of uncertainty (e.g. confidence intervals)
☐	☒	For null hypothesis testing, the test statistic (e.g. *F*, *t*, *r*) with confidence intervals, effect sizes, degrees of freedom and *P* value noted<br>*Give P values as exact values whenever suitable.*
☒	☐	For Bayesian analysis, information on the choice of priors and Markov chain Monte Carlo settings
☒	☐	For hierarchical and complex designs, identification of the appropriate level for tests and full reporting of outcomes
☒	☐	Estimates of effect sizes (e.g. Cohen's *d*, Pearson's *r*), indicating how they were calculated

Our web collection on statistics for biologists contains articles on many of the points above.

Software and code

Policy information about availability of computer code

Data collection	No software was used
Data analysis	1- To assess the burden of infection in the G. mellonella we used GraphPad Prism version 8.4.3 (graphPad Software, LLC).<br>2-The mycetoma grains were visualized on a computer screen using EOS Utility software (Canon Inc.)<br>3- STAR Aligner version 2.7.1a used to map raw sequence reads against the G. mellonella assembly ASM364042v2 and then against M. mycetomatis genome assembly ASM127576v2.<br>4- For the rest of the analysis and plotting figures, we used R software environment for statistical computing and graphics.

For manuscripts utilizing custom algorithms or software that are central to the research but not yet described in published literature, software must be made available to editors and reviewers. We strongly encourage code deposition in a community repository (e.g. GitHub). See the Nature Portfolio guidelines for submitting code & software for further information.

Data

Policy information about availability of data

All manuscripts must include a data availability statement. This statement should provide the following information, where applicable:

- Accession codes, unique identifiers, or web links for publicly available datasets
- A description of any restrictions on data availability
- For clinical datasets or third party data, please ensure that the statement adheres to our policy

Raw sequence data and expression tables used in the analysis have been deposited in the Gene Expression Omnibus (GEO) repository, https://www.ncbi.nlm.nih.gov/geo (accession numbers: GSE213321, GSE213322, GSE213329, GSE213332, and GSE280443). The mass spectrometry proteomics data have been deposited to the ProteomeXchange Consortium via the PRIDE partner repository with the dataset identifier PXD063449 [https://www.ebi.ac.uk/pride/archive/projects/PXD063449].

Research involving human participants, their data, or biological material

Policy information about studies with human participants or human data. See also policy information about sex, gender (identity/presentation), and sexual orientation and race, ethnicity and racism.

Reporting on sex and gender	The study used a model organism the G. mellonella larvae, therefore, this is not applicable to the study.
Reporting on race, ethnicity, or other socially relevant groupings	This is not applicable to the study.
Population characteristics	This is not applicable to the study.
Recruitment	This is not applicable to the study.
Ethics oversight	This is not applicable to the study.

Note that full information on the approval of the study protocol must also be provided in the manuscript.

Field-specific reporting

Please select the one below that is the best fit for your research. If you are not sure, read the appropriate sections before making your selection.

[x] Life sciences [ ] Behavioural & social sciences [ ] Ecological, evolutionary & environmental sciences

For a reference copy of the document with all sections, see nature.com/documents/nr-reporting-summary-flat.pdf

Life sciences study design

All studies must disclose on these points even when the disclosure is negative.

Sample size	To Determine the fungal burden in G. mellonella, a separate group consisting of 15 larvae was infected, and survival was recorded daily for the duration of ten days. At 4, 24, 72 and 168 hours post-infection, the contents of three larvae were pooled and total RNA extracted as detailed in the (methods section).No sample size calculation was carried out, as we are not comparing distinct trait/phenotype. But mapping host and pathogen interaction over time during mycetoma grain formation and determine the pathogen burden in G. mellonell larvae.
Data exclusions	1- G. mellonella larvae were obtained from Terra Equipment Voedseldieren (Cuijk, The Netherlands) and kept in the dark on wood shaving at room temperature until use. Within five days of receipt, larvae of approximately 300-500mg were selected for experimental use. The inclusion criteria of the larvae were established in [PMID: 32267851].<br><br>2- During all experiments, G. mellonella larvae developed to pupa stage were excluded.<br><br>3- Two LQ-ssCAGE (2:15) libraries were excluded due to the poor quality of yielded cDNA. All RNA-seq libraries (15:15), passed known RNA-seq quality check.
Replication	1- To confirm the observation from transcriptomics profiling, for each time point, we have three biological replicates (triplicate). The RNA-seq profiles of all replicates were successful and supported the result reported in the study.<br><br>2-In addition to the experimental design, our analysis were supported by robust statistical techniques as described in the (methods section). high Spearman correlation between the replicates (main fig.2D left) shows the reproducibility of the transcriptomics profiling.<br><br>3.The validation by RT-qPCR of selected differentially expressed genes with known functions, in the infected G. Mellonella libraries confirmed their expression in these samples.
Randomization	Randomization was not applicable in the study. Larvae of approximately 300-500mg were selected for experimental use. The selected larvae were divided over Petri dishes containing 90mm Whatman filter paper and five larvae per dish. 40 µL of the prepared inoculum of M.

	mycetomatis strain MM55 was injected in the last left proleg of the larvae using an insulin 29G U-100 needle (BD diagnostics, Sparsk, USA), resulting in a final concentration of 4 mg fungal biomass/mL. To monitor the course of the infection, a separate group consisting of 15 larvae was infected, and survival was recorded daily for the duration of ten days. During all experiments, Pupa were removed from the equation, and non-infected larvae were included as control. At 4, 24, 72 and 168 hours post-infection, the contents of three larvae were pooled and flash-frozen with liquid nitrogen, followed by mechanical crushing using a pestle and mortar.
Blinding	Blinding were not applicable to this study. All the G. mellonella larvae were healthy when obtained from Terra Equipment Voedseldieren (Cuijk, The Netherlands). G. mellonella larvae were infected with the pathogen and non infected larvae were used as control.

Reporting for specific materials, systems and methods

We require information from authors about some types of materials, experimental systems and methods used in many studies. Here, indicate whether each material, system or method listed is relevant to your study. If you are not sure if a list item applies to your research, read the appropriate section before selecting a response.

Materials & experimental systems

n/a	Involved in the study
☒	☐ Antibodies
☒	☐ Eukaryotic cell lines
☒	☐ Palaeontology and archaeology
☐	☒ Animals and other organisms
☒	☐ Clinical data
☒	☐ Dual use research of concern
☒	☐ Plants

Methods

n/a	Involved in the study
☒	☐ ChIP-seq
☒	☐ Flow cytometry
☒	☐ MRI-based neuroimaging

Animals and other research organisms

Policy information about studies involving animals; ARRIVE guidelines recommended for reporting animal research, and Sex and Gender in Research

Laboratory animals	The study did not involve laboratory animals .
Wild animals	The study did not involve wild animals.
Reporting on sex	Sex-based analysis was not applicable to the study, we are studying mycetoma grain formation and development in a model organism the G. mellonell larvae.
Field-collected samples	The study did not involve samples collected from the field.
Ethics oversight	No ethical approval or guidance was required, we used a model organism free of the legal/ethical restrictions associated with the use of mammals.

Note that full information on the approval of the study protocol must also be provided in the manuscript.

Plants

Seed stocks	*Report on the source of all seed stocks or other plant material used. If applicable, state the seed stock centre and catalogue number. If plant specimens were collected from the field, describe the collection location, date and sampling procedures.*
Novel plant genotypes	*Describe the methods by which all novel plant genotypes were produced. This includes those generated by transgenic approaches, gene editing, chemical/radiation-based mutagenesis and hybridization. For transgenic lines, describe the transformation method, the number of independent lines analyzed and the generation upon which experiments were performed. For gene-edited lines, describe the editor used, the endogenous sequence targeted for editing, the targeting guide RNA sequence (if applicable) and how the editor was applied.*
Authentication	*Describe any authentication procedures for each seed stock used or novel genotype generated. Describe any experiments used to assess the effect of a mutation and, where applicable, how potential secondary effects (e.g. second site T-DNA insertions, mosiacism, off-target gene editing) were examined.*
